# Characterization of SCO4439, a D-alanyl-D-alanine carboxypeptidase involved in spore cell wall maturation, resistance, and germination in *Streptomyces coelicolor*

**DOI:** 10.1038/srep21659

**Published:** 2016-02-12

**Authors:** Beatriz Rioseras, Paula Yagüe, María Teresa López-García, Nathaly Gonzalez-Quiñonez, Elisa Binda, Flavia Marinelli, Angel Manteca

**Affiliations:** 1 Área de Microbiología, Departamento de Biología Funcional and IUOPA, Facultad de Medicina, Universidad de Oviedo, 33006 Oviedo, Spain; 2 Department of Biotechnology and Life Sciences, University of Insubria, via J. H. Dunant 3, 21100 Varese, Italy; 3 “The Protein Factory” Research Center, Politecnico of Milano, ICRM CNR Milano and University of Insubria, 21100 Varese, Italy

## Abstract

This work contributes to the understanding of cell wall modifications during sporulation and germination in *Streptomyces* by assessing the biological function and biochemical properties of SCO4439, a D-alanyl-D-alanine carboxypeptidase (DD-CPase) constitutively expressed during development. SCO4439 harbors a DD-CPase domain and a putative transcriptional regulator domain, separated by a putative transmembrane region. The recombinant protein shows that DD-CPase activity is inhibited by penicillin G. The spores of the *SCO4439::Tn5062* mutant are affected in their resistance to heat and acid and showed a dramatic increase in swelling during germination. The mycelium of the *SCO4439::Tn5062* mutant is more sensitive to glycopeptide antibiotics (vancomycin and teicoplanin). The DD-CPase domain and the hydrophobic transmembrane region are highly conserved in *Streptomyces*, and both are essential for complementing the wild type phenotypes in the mutant. A model for the biological mechanism behind the observed phenotypes is proposed, in which SCO4439 DD-CPase releases D-Ala from peptidoglycan (PG) precursors, thereby reducing the substrate pool for PG crosslinking (transpeptidation). PG crosslinking regulates spore physical resistance and germination, and modulates mycelium resistance to glycopeptides. This study is the first demonstration of the role of a DD-CPase in the maturation of the spore cell wall.

Streptomycetes are mycelial microorganisms characterized by their complex developmental cycles, including programmed cell death (PCD) and hyphae differentiation, which leads to aerial mycelium formation and sporulation^1^,^2^. Streptomycetes are important industrial bacteria producing approximately two-thirds of clinical antibiotics, as well as a large number of eukaryotic cell differentiation inducers and inhibitors^3^. Most of these bioactive compounds are specialized metabolites^4^, the production of which is regulated, at least in part, by hyphal differentiation^5^. *Streptomyces* development, is activated by extracellular signals, including nutritional stimuli or cell density (quorum sensing), and is regulated by complex signaling pathways that are only partially known^5^,^6^,^7^. The best-characterized stages of *Streptomyces* development are those related to aerial mycelium and sporulation. Several key regulatory networks controlling these stages have been characterised (bald, sky or white pathways, reviewed in Flärdh and Buttner^1^). Despite this, the regulation of aerial mycelium and sporulation remains incompletely understood, and new genes and proteins regulating these important processes, are still being discovered^8^. Stages preceding aerial mycelium, including spore germination and differentiation in liquid non-sporulating cultures, are even less characterized and comprehended^2^,^9^.

D-alanyl-D-alanine carboxypeptidases (DD-CPases) are members of the penicillin binding proteins (PBPs), a family of proteins inhibited by ß-lactam antibiotics involved in peptidoglycan (PG) synthesis and remodelling. The PBPs constitute a family of acyltransferases with a common evolutionary origin and a common substrate (the D-Ala-D-Ala dipeptides present in the pentapeptide stems of PG precursors), that are collectively known as DD-peptidases. DD-peptidases include transglycosylases, which catalyze the polymerization of glycan chains composed of N-acetylglucosamine (NAG) and N-acetylmuramic acid (NAM); DD-CPases, which remove the terminal D-alanine from muramyl pentapeptide; transpeptidases, which catalyze the cross-link formation between one D-Ala of one PG strand and one amino acid of the other strand; and endopeptidases, which cleave the cross-linked peptide side-chains^10^. The DD-peptidases are classified on the basis of their molecular mass, amino acid sequence and enzyme activity at high molecular mass (HMM) and low molecular mass (LMM)^11^,^12^,^13^. HMM DD-peptidases are usually bifunctional transglycosylases/transpeptidases (also classified as class A), or monofunctional transpeptidases (class B) anchored to membranes. LMM DD-peptidases are monofunctional carboxypeptidases or endopeptidases, and the majority are also anchored to membranes (class C). LMM DD-peptidases are usually not essential, and they are not found in most studies and reviews on PBPs^13^.

Most bacteria have multiple PBPs with varying degrees of redundant activity. A phylogenetic analysis revealed that Actinobacteria have an average of five HMM DD-peptidases per genome; this number doubled only in streptomycetes (13 DD-peptidases in the case of *S. coelicolor*)^13^. Many DD-peptidases belong to the SEDS (shape, elongation, division, and sporulation) clusters of genes involved in PG synthesis and remodeling and are essential for growth, cell division, and cellular viability. DD-peptidases that are not included in the SEDS clusters are considered dispensable for growth and viability^10^, and their biological function remains poorly investigated. *Streptomyces coelicolor* harbors four SEDS clusters^14^, that include four transpeptidases (SCO2090, SCO2608, SCO3847 and SCO5301).

This work contributes characterizing the biological function of the largely ignored redundant and non-essential LMM DD-peptidases by studying the activity and role of *SCO4439*, a gene encoding a putative DD-CPase. *SCO4439* is a very unusual DD-CPase that is highly conserved in streptomycetes and is therein fused with a putative transcriptional regulator domain (see below). *SCO4439* was found to be slightly over-expressed during the aerial mycelium and sporulation stages^15^, however, its biological role remained unclear.

## Results

### Mutation of *SCO4439* affects spore swelling during germination, increases spore resistance to acid/heating and reduces the glycopeptide resistance

**Figure 1:**
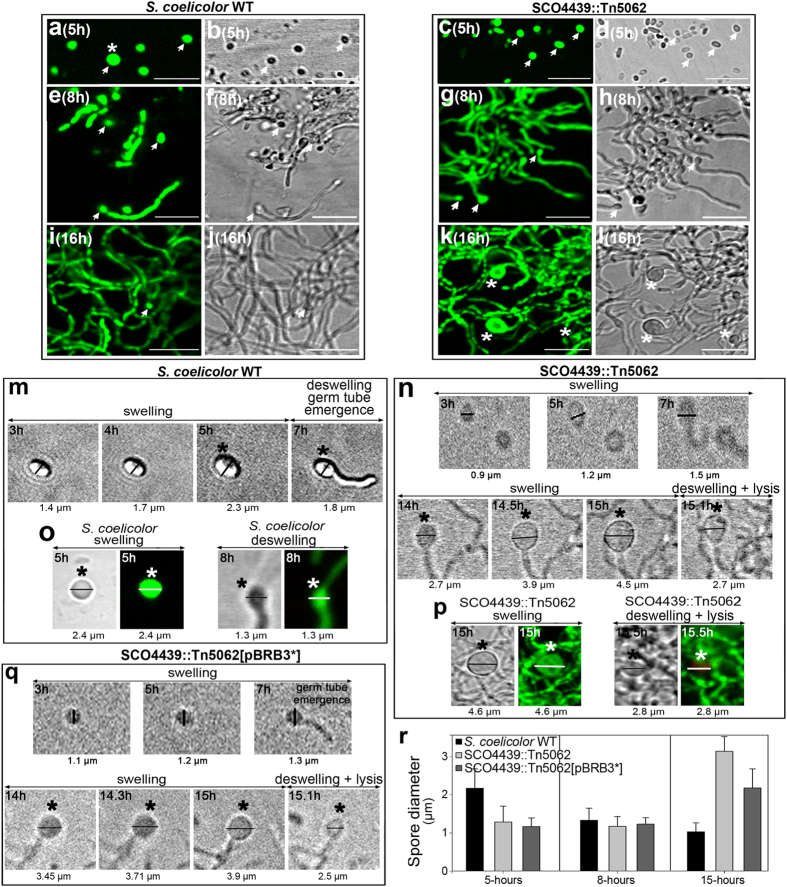
Analysis of the germination stages in *S. coelicolor* wild type and in *S. coelicolor SCO4439::Tn5062* mutant. (**a**–**l**) Confocal laser-scanning fluorescence microscopy analysis (SYTO9/PI staining) of the *S. coelicolor* wild type (left panel) and *SCO4439::Tn5062* mutant (right panel). The same samples were observed using the fluorescence (left pictures) or interference contrast modes (right pictures). Bars indicate 8 μm (**m,n**) Time-lapse confocal microscopy (interference contrast mode) of the germination of spores from the wild type and of the *SCO4439::Tn5062* mutant, respectively. Spore diameters are indicated. (**o,p**) Confocal laser-scanning fluorescence microscopy analysis (SYTO9/PI staining) and interference contrast images of spore swelling and spore deswelling stages in the wild type and *SCO4439::Tn5062* mutant, respectively. (**q**) Time-lapse confocal microscopy of the germination of spores from the *SCO4439::Tn5062* mutant harboring the SCO4439* mutated gene (SCO4439::Tn5062[pBRB3*] strain). Time-lapse experiments were limited to 12 hours (see Methods for details). Arrows indicate spores. Asterisks indicate swelled spores. (**r**) Quantification of the spore diameters (average ± SD) of the wild type, *SCO4439::Tn5062* mutant, and *SCO4439::Tn5062*[pBRB3*] strain at 5, 8 and 15 hours.

Cosmid D6.2.B06_046 harboring a copy of *SCO4439* interrupted by Tn5062 was used to obtain the *S. coelicolor SCO4439::Tn5062* mutant using the methodology developed by Fernández-Martínez *et al*.^16^. The early stages of *SCO4439::Tn5062* mutant spore germination were similar to those in the *S. coelicolor* wild strain (Fig. 1a–h); however *SCO4439::Tn5062* mutant showed a clear and distinctive phenotype at later stages, consisting of a dramatic increase in spore swelling (Fig. 1k,l) with respect to the wild-type strain (Fig. 1i,j).

The kinetics of spore germination in the *S. coelicolor* wild type and in the *SCO4439::Tn5062* mutant were studied by using time-lapse confocal microscopy (Fig. 1m,n and Supplementary Movies 1 and 2). The spores of the *S. coelicolor* parental strain swelled until they reached a diameter of 2.3 ± 0.4 μm (5-hour culture), before the emergence of the germ tube, which coincided with a deswelling and a consequent reduction of the spore diameter (from 2.3 ± 0.4 μm at 5-hours to 1.2 ± 0.4 μm at 8-hours) (Fig. 1m,r). At early time points, the swelling of the spores of the *SCO4439::Tn5062* mutant was slower than the swelling of the spores of the wild strain (compare 5 hour-time in Fig. 1m,n), but they continued to swell after the emergence of the germ tube, reaching a diameter that was the double that of the wild type spores (3.2 μm ± 0.4 μm) before deswelling and reducing the spore diameter (Fig. 1n,r). Another difference between the wild type and the *SCO4439::Tn5062* mutant was that, after germination and deswelling, the cell membrane permeability inside the spores remained intact in the wild type strain (SYTO9 staining, Fig. 1o), but not in the *SCO4439::Tn5062* mutant (PI staining, Fig. 1p). The increase in spore swelling affected 100% of the *SCO4439::Tn5062* mutant spores at 15–18 hours (average diameter of 3.1 ± 0.4 μm, Fig. 1r).

**Figure 2:**
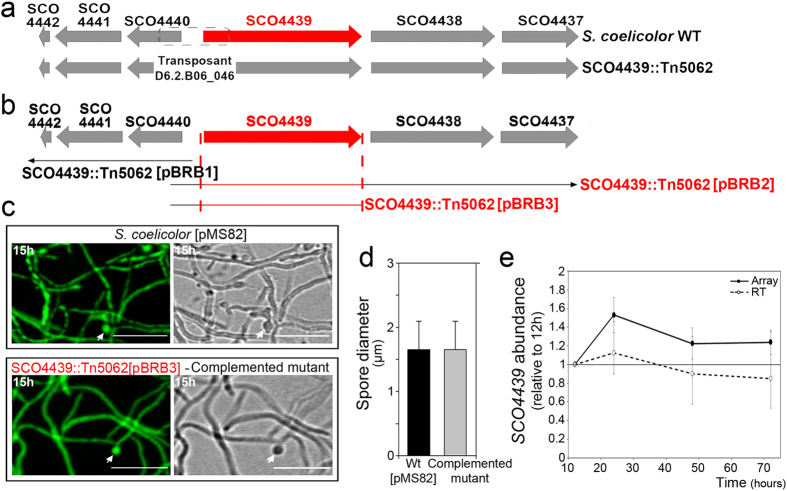
Complementation of the wild-type phenotype in the *SCO4439::Tn5062* mutant, and *SCO4439* gene expression during development. (**a**) Scheme of the *SCO4437*-*SCO4442* region in the wild type and the *SCO4439::Tn5062* mutant. The dashed line indicates the chromosome deletion generated by the transposon insertion. (**b**) Scheme illustrating the fragments introduced into the mutant strain using plasmids pBRB1, pBRB2 and pBRB3. Fragments that complemented the phenotype in the mutant strain are highlighted in red. (**c**) Confocal laser fluorescence microscopy analysis of the *S. coelicolor* wild-type strain harboring pMS82 (control), and the *S. coelicolor SCO4439::Tn5062* mutant strain harbouring pBRB3 (complementation of the phenotype is also observed with pBRB2 but not with pBRB1). Samples were observed using the fluorescence (left pictures) or interference contrast modes (right pictures). Arrows indicate spores. (**d**) Quantification of spore diameters (average ± SD) in the wild type harboring pMS82 and in the *SCO4439::Tn5062* mutant harboring pBRB3. (**e**) *SCO4439* gene abundance at 12, 24, 48 hours (aerial mycelium) and 72 hours (spores). Data represent the fold change with respect to the 12 hour time point (qRT-PCR and microarray values). Microarray data are from Yagüe *et al*.^2^.

Contrary to expectations^16^, DNA sequencing demonstrated that the insertion of Tn5062 into cosmid D6.2.B06_046 generated a deletion. Tn5062 was inserted at position 640 of *SCO4439* and 669 of *SCO4440*, generating a loss of 1,641 bp, which affected the 5′-terminus of *SCO4439* and most of the *SCO4440* open reading frame (ORF) (schematized in Fig. 2a). The deletion of the 5′-terminus of *SCO4439* may affect the expression of *SCO4437* and *SCO4438* (both located downstream of *SCO4439*), whereas the deletion of *SCO4440* may affect the expression of *SCO4441* and *SCO4442* (Fig. 2a). To identify the gene responsible for the observed phenotype, plasmid pMS82^17^ was used to introduce different fragments of the *SCO4437*-*SCO4442* chromosomal region, into the *SCO4439::Tn5062* mutant strain (schematized in Fig. 2b). The only DNA fragments complementing the wild-type phenotype were those including *SCO4439,* as was the case for plasmid pBRB3 (compare Fig. 2c with Fig. 1k,l) (Supplementary Movie 3). Similar results were obtained in the *SCO4439::Tn5062* mutant harboring plasmid pBRB2 but not in the mutant strain harboring plasmid pBRB1 (data not shown).

As introduced above, the *SCO4439* gene was previously reported to be slightly over-expressed during aerial mycelium and sporulation in microarray-based transcriptomic analyses; however differences in expression were too low to be considered significant^15^ (Fig. 2e). In this work, RT-PCR analyses confirmed the expression pattern of *SCO4439* (Fig. 2e).

**Figure 3:**
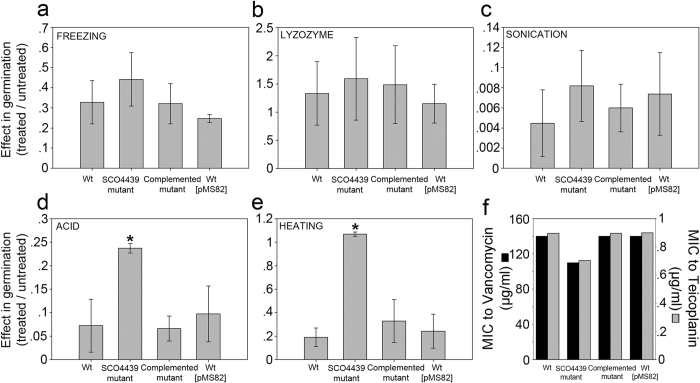
Spore resistance to physicochemical stresses and mycelium resistance to glycopeptides (vancomycin and teicoplanin). (**a**) Spore resistance to freezing. (**b**) lysozyme. (**c**) sonication. (**d**) acid. (**e**) heating. (**f**) Minimum inhibitory concentrations of vancomycin and teicoplanin towards *S. coelicolor*. The (1) effect on germination of the treated spores with respect to the untreated spores, and the (2) MIC values were estimated for the *S. coelicolor* wild-type strain (wt), *SCO4439::Tn5062* mutant (SCO4439 mutant), *SCO4439::Tn5062* complemented with pBRB3 (complemented mutant) and the control wild type strain harboring pMS82 (wt pMS82). Note that lysozyme treatment increased germination in all strains. Asterisks indicate a significant increase in resistance to acid and heating in the mutant strain. Percentages of germination are the average ± SD of three replicates; MIC values were estimated using three biological replicates; SD = 0.

The spore resistance profiles of the *SCO4439::Tn5062* mutant and the wild type strains were compared (Fig. 3). Lysozyme treatment increases germination, and sonication kills 99% of the spores in the *S. coelicolor* wild strain. The *SCO4439* mutation did not affect resistance to the lysozyme, to sonication or to freezing (Fig. 3a–c) but increased fivefold the spore resistance to acid and heating compared with the parental strain and the *SCO4439::Tn5062* complemented mutant (Fig. 3d,e).

Mycelium resistance to glycopeptides (vancomycin and teicoplanin) was reduced in the *SCO4439::Tn5062* mutant (minimum inhibitory concentrations of 110 and 0.7 μg/ml, respectively), in respect to the *S. coelicolor* wild type strain (minimum inhibitory concentrations 140 and 0.9 μg/ml) (Fig. 3f).

### SCO4439 harbors two protein domains separated by a putative hydrophobic transmembrane region

**Figure 4:**
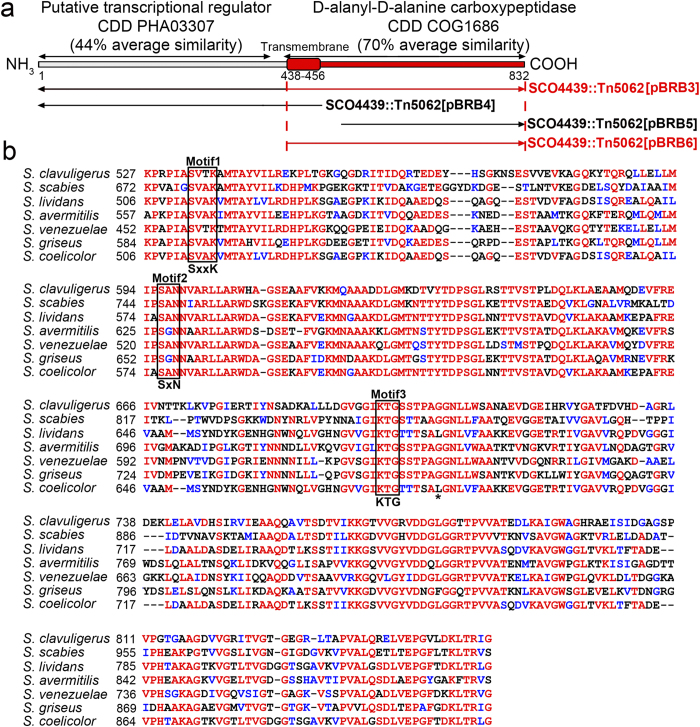
Structure of SCO4439 and orthologous proteins in the *Streptomyces* genus. (**a**) Scheme indicating the structure of the SCO4439 protein. Conserved database domain (CDD) references and their average similarities in the *Streptomyces* genus are indicated. *SCO4439* fragments introduced in plasmids pBRB3, pBRB4, pBRB5 and pBRB6 are shown schematically. Fragments that complemented the phenotype in the mutant strain are highlighted in red. (**b**) Sequence alignment of the DD-CPase domain of SCO4439 (*S. coelicolor*) and their orthologs in other model streptomycetes. Conserved “SxxK”, “SxN” and “KTG” motifs that characterize the “SxxK” superfamily of DD-peptidases are indicated. An asterisk indicates the Leu_684_ whose replacement by Pro partially blocks complementation of the wild type phenotype in the *SCO4439::Tn5062* mutant.

*SCO4439* encodes a multi-domain protein harboring a DD-CPase (conserved domain database accession COG1686), and a putative transcriptional regulator (conserved domain database accession PHA03307) (Fig. 4a). According to the Phobius software prediction (http://phobius.sbc.su.se/), these two domains are separated by a putative transmembrane region (Fig. 4a). The DD-CPase domain and the putative hydrophobic transmembrane domain are highly conserved in the *Streptomyces* genus (70% average similarity in *Streptomyces*) and actinomycetes in general (data not shown). The conservation of the transcriptional regulatory domain is substantially lower (44% average similarity in *Streptomyces*), and this domain is not present in other actinomycetes.

Different fragments of the *SCO4439* gene were introduced into the *SCO4439::Tn5062* mutant strain, using pMS82 as the integrative carrier vector (see Materials and Methods for details and scheme in Fig. 4a). The only fragments that complemented the wild-type phenotype were those containing both, the transmembrane and DD-CPase domains (data not shown).

Amino acid sequence alignment of the *S. coelicolor* DD-CPase domain (Fig. 4b) showed an overall high identity with the orthologous proteins from the six model *Streptomyces* strains analyzed, including the triad of “SxxK”, “SxN” and “KTG” motifs that characterize the “SxxK” superfamily of penicillin-binding DD-peptidases^11^. Interestingly, a replacement of Leu_684_ by Pro (randomly generated by PCR, see Methods) partially blocked the phenotype complementation in spore swelling in the *SCO4439::Tn5062* mutant (Fig. 1q). As discussed below, the maximum spore-swelling of the *SCO4439::Tn5062* mutant strain harboring the mutated SCO4439* gene (*SCO4439::Tn5062* [pBRB3*] strain) was 3.9 μm (Fig. 1q), which is an intermediate value between the wild type (2.3 μm) and the *SCO4439:Tn5062* mutant (4.5 μm) spore diameters (Fig. 1m,n,q,r; Supplementary Movie 4).

### SCO4439 carboxypeptidase activity

**Figure 5:**
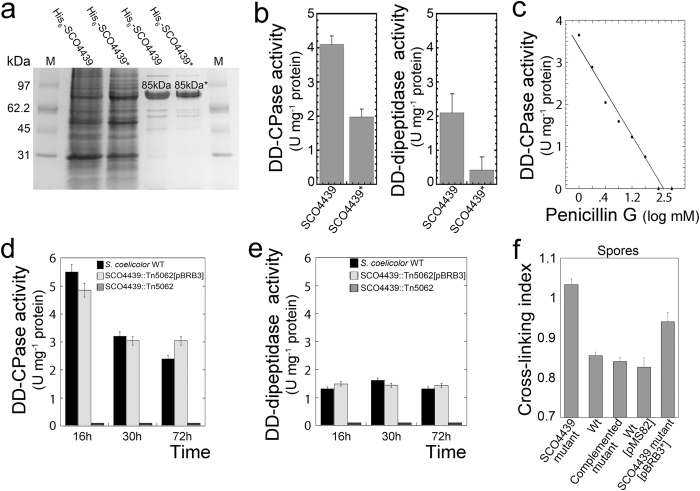
Purification of recombinant His_6_-SCO4439/ His_6_-SCO4439*, SCO4439 activity and cellular localization. (**a**) Coomassie-stained SDS-PAGE gel of overproduced His_6_-SCO4439 and His_6_-SCO4439* (substitution of Leu_684_ for Pro_684_). M, molecular weight markers. Lane 1, *E. coli* JM109 producing His_6_-SCO4439 (45 μg). Lane 2, *E. coli* JM109 producing His_6_-SCO4439* (45 μg). Lane 3, purified His_6_-SCO4439 (4 μg). Lane 4 purified His_6_-SCO4439* (4 μg). (**b**) DD-CPase and DD-dipeptidase activities of His_6_-SCO4439 and His_6_-SCO4439*. Enzyme activity values (units per mg of pure recombinant protein) are the average ± SD of three replicates. (**c**) His_6_-SCO4439 penicillin inhibition curve. (**d**,**e**) DD-CPase and DD-dipeptidase activity (units per mg of total protein) detected in insoluble fractions (membrane and cell wall debris) of *S. coelicolor* wild type and of *SCO4439::Tn5062* mutant strains. (**f**) PG crosslinking in the spores of the *SCO4439::Tn5062* mutant (SCO4439 mutant), *S. coelicolor* wild type (wt), *SCO4439::Tn5062* complemented with pBRB3 (complemented mutant), the control wild type strain harboring pMS82 (wt pMS82), and the *SCO4439::Tn5062* mutant complemented with pBRB3* (SCO4439 mutant [pBRB3*]). Values are the means ± SD of two biological replicates, and four technical replicates.

The SCO4439 protein and its mutated version SCO4439* (mutation replacing Leu_684_ with Pro_684_, see above) were over-expressed in *E. coli*, and purified using His-tag affinity chromatography (Fig. 5a). The identity of the overproduced proteins was confirmed via peptide mass fingerprinting (data not shown).

The enzymatic activities of the purified SCO4439 and SCO4439* were assayed on D-Ala-D-Ala dipeptide and on the tripeptide N_α_,N_ε_-diacetyl-l-Lys-D-Ala-D-Ala, which mimics the terminal portions of the PG pentapeptide precursors^17^,^18^. SCO4439 and SCO4439* cleaved the D-Ala from the tripeptide and, to a lesser extent, from the dipeptide D-Ala-D-Ala. Thus, they showed a significantly higher DD-CPase activity than DD-dipeptidase activity (Fig. 5b). The enzyme activity of the mutated SCO4439* was half that of the non-mutated protein (Fig. 5b and discussed below). As expected, due to the presence of the canonical Ser-x-x-Lys motif present in SCO4439, the enzyme activity was inhibited by penicillin G (IC_50_ of 1 mM, Fig. 5c). As a control, no DD-CPase/DD-dipeptidase activity was detectable in the *E.coli* host transformed with the empty expression vectors (data not shown).

The DD-CPase/DD-dipeptidase activity of SCO4439 was then assayed in extracts from wild type *S. coelicolor,* the *SCO4439::Tn5062* mutant, and the complemented mutant. The DD-CPase/DD-dipeptidase activity was always detectable in insoluble fractions (membranes and cell wall debris) from the *S. coelicolor* wild-type strain and the complemented mutant, but not in those from the *S. coelicolor SCO4439::Tn5062* mutant (Fig. 5d,e). There was no detectable activity in the cytosolic fractions from any of the two strains (data not shown). Interestingly, the specific DD-CPase enzymatic activity was slightly higher at the early time points after germination (16 hours) than in the substrate (30 hours) or aerial (72 hours) mycelium stages (Fig. 5d). As expected, incubation of the insoluble fractions with penicillin G abolished the enzyme’s activity (data not shown).

The spore PG crosslinking index (ratio between cross-linked glycine and total glycine) was estimated by adapting the 1-fluoro-2,4-dinitrobenzene (FDNB) method originally described for *Bacillus*^19^ to *Streptomyces* (see Methods for details) (Fig. 5f). As discussed below, the spores of the *S. coelicolor SCO4439::Tn5062* mutant had 20% more crosslinking than the spores of the wild type strain. Interestingly, the PG crosslinking of the *SCO4439::Tn5062* harboring the mutated SCO4439* gene (*SCO4439::Tn5062* [pBRB3*]) were at an intermediate level in PG crosslinking between the *SCO4439::Tn5062* mutant and the wild type strain (10% more crosslinking than the spores of the wild type strain; Fig. 5f).

### PG synthesis during spore swelling and germination

**Figure 6:**
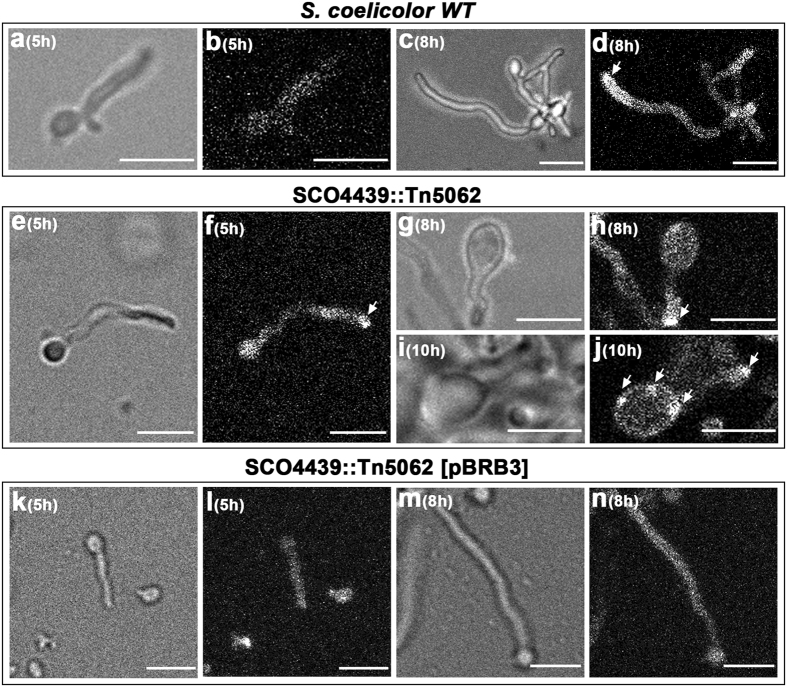
Nascent-PG synthesis during germination. (**a**–**d**) *S. coelicolor* wild type. (**e**–**j**) *SCO4439::Tn5062* mutant. (**k**–**n**), (*SCO4439::Tn5062*[pBRB3] complemented strain. GYM liquid cultures were stained with BODIPY-vancomycin, and observed at the confocal microscope. The interference contrast mode (left pictures) and fluorescent images (right pictures) are shown. Arrows indicate nascent PG. Developmental time points are indicated. Scale bars represent 4 μm.

Peptidoglycan synthesis during spore germination and swelling was analyzed using fluorescent BODIPY-vancomycin^20^ (Fig. 6). BODIPY-vancomycin preferentially stains nascent PG, the staining of which protrudes above the areas in which there is no PG synthesis^20^. No PG synthesis occurred during the spore germination and swelling early stages in the wild and complemented strains, at least in the amount detectable by BODIPY-vancomycin staining (Fig. 6). Only at the latest stages of spore swelling, some areas of PG synthesis become detectable in the swelled spores of the mutant strain (Fig. 6j).

## Discussion

The aim of this work was the characterization of SCO4439, a DD-CPase constitutively expressed during development (Fig. 2e), and whose enzymatic activity is present during all developmental stages analyzed (Fig. 5d). The best characterized DD-peptidases are those belonging to the SEDS genes involved in PG synthesis/remodeling which are essential for growth, cell division, and viability. However, with very few exceptions, the biological role of other redundant DD-peptidases, not included in the SEDS clusters, such as SCO4439, remains unknown^19^,^21^. SCO4439 is not essential for growth; however its mutation resulted in a dramatic increase in both spore resistance to acid/heating and swelling during germination.

DD-CPases are usually anchored to cell membranes at their N-terminus, and their active sites are exposed to the periplasmic space in which they catalyze the final stages of cell wall biosynthesis^11^. SCO4439 is very unusual, because in addition to the DD-CPase domain, it harbors an additional putative transcriptional regulator domain. Other DD-CPases, including most HMM DD-CPases, are multi domain proteins and harbor glycosyl transferase domains involved in cell wall maturation^11^. However, to our knowledge, SCO4439 is the first DD-CPase that is associated with a putative transcriptional regulatory domain. SCO4439 has a high molecular mass of 84 kDa, resulting in its classification as an HMM DD-CPase. However, the DD-peptidase domain of SCO4439 is homologous to LMM DD-peptidases and exhibits the DD-CPase activity that is peculiar to LMM DD-peptidases^11^. The putative transmembrane domain of SCO4439 is located in the middle of the protein, separating the DD-CPase and the putative transcriptional regulator domains, which is also unusual. The (1) Phobius prediction of the protein structure, (2) the presence of DD-CPase activity only in *S. coelicolor* insoluble fractions (membranes and cell wall debris) (Fig. 5d) and (3) the presence of the essential nature of the transmembrane domain for biological activity (Fig. 4a) suggest that the DD-CPase domain is exposed to the periplasmic space, whereas the putative transcriptional regulatory domain is likely located on the cytosol side of the cell membrane. Further work is required to understand the biological function, if any, of the putative transcriptional regulatory domain located at the N-terminus of SCO4439. The presence of this domain in all SCO4439 orthologs suggests that it may have a relevant function (Fig. 4). In contrast, its relatively low conservation (44% average similarity), its exclusive occurrence in streptomycetes, and finally, its unessential role in complementing the spore germination and resistance phenotypes in the *SCO4439::Tn5062* mutant may indicate that this domain has not an essential function.

**Figure 7:**
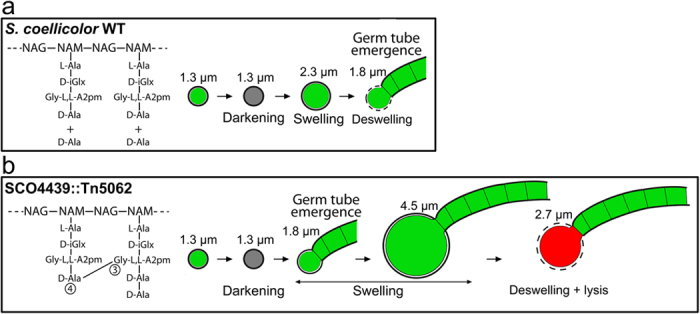
Model for the biological function of SCO4439. (**a**) *S. coelicolor* wild type. (**b**) *SCO4439::Tn5062.* Classical germination stages: darkening, swelling and germ tube emergence^24^. The proposed spore deswelling stage is indicated. Green illustrates the membrane-intact cells (SYTO 9 staining); red indicates propidium iodide (PI) permeable cells (lysis). D-iGlx, D-*iso*-glutamine or D-*iso*-glutamic acid. See text for details.

Spore germination comprises a succession of distinctive steps that were organized by Hardisson *et al*.^22^ into three stages: darkening, swelling, and germ tube emergence (Fig. 7). The biomolecular mechanisms controlling these stages remain poorly characterized^23^,^24^,^25^. There are two examples of proteins known to be involved in *Streptomyces* spore germination. NepA was described as a structural cell wall protein involved in the maintenance of spore dormancy in *S. coelicolor*^26^. SsgA was identified as a protein marking cell-wall sites in which germination takes place^27^. The phenotype of the *SCO4439::Tn5062* mutant observed in this work indicates the existence of a new stage that includes the deswelling of the spores once they cannot resist further swelling. The occurrence of this stage was demonstrated via time-lapse microscopy in both the wild type and the *SCO4439::Tn5062* mutant (Supplementary Movies 1 and 2; Fig. 1m,n). In the wild-type strain, spore deswelling coincided with germ tube emergence, whereas the spores of the *SCO4439::Tn5062* mutant continued to swell after the emergence of the germ tube (Fig. 7). In the partially complemented *SCO4439::Tn5062* mutant (the strain complemented with the SCO4439* mutated gene), spore swelling persisted also after germ tube emergence, but the maximum swelling was lower than in the mutant (maximum spore diameter of 3.9 μm vs. the 4.6 μm reached in the mutant; Fig. 1p,q; Supplementary Movies 2 and 4).

The proposed biomolecular model to account for the observed phenotypes in the *SCO4439::Tn5062* mutant is schematized in Fig. 7. Accordingly, a deficiency in SCO4439 DD-CPase increments the pool of available transpeptidase substrates (PG pentapeptides), thereby enhancing the activity of these enzymes and promoting the formation of PG crosslinks. Spores with highly crosslinked PG are more resistant to heating and acid and swell more slowly at early germination time points, but they can reach greater dimensions before lysing due to osmotic shock (Fig. 7). The mutation in SCO4439 that replaces Leu_684_ with Pro_684_, halved the native DD-CPase activity, and when the SCO4439* gene was introduced into the *SCO4439::Tn5062* mutant, the phenotype was only partially restored. This demonstrates that spore swelling during germination is proportional to SCO4439 DD-CPase activity. The model proposed in Fig. 7 would also be valid for new PG synthetized in the *SCO4439::Tn5062* mutant after spore germination, at the latest stages of the swelling, which will have more cross-linking than the wild strain.

*Streptomyces* mutants created in germination such as *SCO4439::Tn5062*, represent a key tool that provides insight into this process. Up to now, the osmotic mechanism controlling spore swelling is largely unknown. Germ tube emergence is marked by SsgA^27^, and uncharacterized lytic enzymes regulate the splitting of the spore covers at this germination point^28^. Spore swelling may facilitate the emergence of the germ tube^22^, and germ tube emergence coincides with the end of the swelling in the *S. coelicolor* wild type strain (Fig. 1m). The dramatic swelling of the spores in the *SCO4439::Tn5062* mutant after germ tube emergence suggests that the high osmotic pressure in the spore cytoplasm feeds this swelling. In the weaker spores of the wild-type strain (low PG crosslinking), spore swelling culminate with the emergence of the germ tube. However, spores of the *SCO4439::Tn5062* mutant have higher PG crosslinking, and the swelling continues after the emergence of the germ tube. In this scenario, the cells likely still detect the high osmotic pressure that in normal conditions would indicate the absence of germination, thus they increase swelling and osmotic pressure to facilitate germination (Fig. 7). Further work is required to fully comprehend this phenomenon. Interestingly, some studies have already suggested a role for PBPs in spore germination in other sporulating bacteria such as *Bacillus.* Neyman and Buchanan^29^ and Murray *et al*.^30^ described how some DD-peptidases are expressed differentially during *Bacillus* sporulation and germination, and Buchanan and Gustafson^31^ showed that *dacB* mutants produce spores with unusual resistance to chemicals and heating in *Bacillus*.

The lack of the DD-CPase activity in *SCO4439::Tn5062* mutant strain increases the pool of PG pentapeptide, the terminal D-Ala-D-Ala dipeptides of which are the molecular target of glycopeptide antibiotics^32^. Consequently, the mycelium of the *SCO4439::Tn5062* mutant was more sensitive to vancomycin and teicoplanin than the mycelium of the wild-type strain (Fig. 3f). *S. coelicolor* resistance to vancomycin (but not to teicoplanin), was described to be due to the canonical set of *vanRSHAX* genes induced by vancomycin (but not by teicoplanin) that are responsible for replacing the terminal D-Ala-D-Ala dipeptides with the resistant D-Ala-D-Lac dipeptides^33^,^34^. Recent work demonstrated that other enzymes (VanY-like) contribute to glycopeptide resistance in actinomycetes by removing the last D-Ala from the PG-pentapeptide precursors^34^,^35^. Interestingly, these enzymes are membrane-associated LMW DD-CPases with a minor activity on dipeptides and are in some cases inhibited by β-lactams^35^,^36^.

The *SCO4439* DD-CPase gene is constitutively expressed (Fig. 2e), whereas the specific DD-CPase enzymatic activity decreases during development (from 5 U/mg protein at 16 hours, to 3 U/mg protein at 72 hours) (Fig. 5d). This can be a consequence, that, at later time points, most of the mycelium suffers a programmed cell death^1^,^2^ disrupting cell membrane integrity and experiencing an increasing proteolytic activity. Loss of DD-CPase activity may be due to the increasing protein instability in the above conditions. Anyhow, the occurrence of other specific post-translational modifications regulating the DD-CPase activity cannot be ruled out.

Overall, this work demonstrates that the SCO4439 DD-CPase regulates the proportion of PG crosslinking in the spore cell walls, a process that is critical for the regulation of spore germination. The *SCO4439* DD-CPase gene is constitutively expressed, and its activity is present in the *Streptomyces* vegetative hyphae. However, its biological role in the mycelium (beyond the increase of resistance to glycopeptide antibiotics) remains unknown. Knowledge of the biological role of the genes involved in antimicrobial resistance is important to understand the evolution of resistance in nature.

## Methods

### Bacterial strains and media

Bacterial strains are listed in Table 1. *Streptomyces coelicolor* M145 was the reference strain and was used to generate the mutants. Petri dishes (8.5 cm) with 25 ml of solid GYM medium (glucose, yeast/malt extract)^37^ were covered with cellophane disks, inoculated with 100 μl of a fresh spore suspension (1 × 10^7^ viable spores/ml), and incubated at 30 °C. Spores were obtained from SFM solid cultures^38^.

*Escherichia coli* strains were grown at 37 °C in solid (2% agar) or liquid 2xTY medium^39^ supplemented with the appropriate antibiotics (Table 1).

### Disruption of *SCO4439*

The transposon insertion single-gene knockout library created by Prof. P. Dyson’s research group^16^ was used for mutagenesis of *SCO4439*. Cosmid D6.2.B06 was used to construct the *SCO4439::Tn5062* mutant strain (Table 1). Gene disruption was carried out by obtaining double cross-overs via conjugation using *E. coli* ET12567/pUZ8002 as a donor strain and following the protocol described in Kieser *et al*.^38^. Mutant strains were confirmed using Southern blotting with chromosomal DNA digested with *SalI.* Southern hybridization was carried out using established procedures with the digoxigenin-labeled 3442-bp Tn5062 *PvuII* fragment from plasmid pQM5062^40^ as a probe.

### Complementation of *SCO4439::Tn5062* mutation

The integrative plasmid pMS82^41^ was used to introducedifferent fragments from the *SCO4437*-*SCO4442* chromosomal region into the *SCO4439::Tn5062* mutant. Fragments were amplified via PCR using Phusion High-Fidelity DNA Polymerase (Thermo), and were then cloned into pCR™-Blunt II-TOPO^®^. The sequences were checked via DNA sequencing using the M13 universal primers prior to subcloning them into pMS82. The following plasmids were constructed (Table 1): pBRB1 containing the *SCO4440*-*SCO4442* fragment amplified with primers BRB1F/BRB1R; pBRB2 containing the *SCO4437*-*SCO4439* fragment amplified with primers BRB2F/BRB2R; pBRB3 containing *SCO4439* amplified with primers BRB2F/BRB3R. One of the amplified *SCO4439* fragments cloned in pCR™-Blunt II-TOPO^®^ had a mutation generated during the PCR that replaced Leu_684_ with Pro; this mutation was also cloned into pMS82 generating plasmid pBRB3*. In all cases, primers were designed to hybridize at least 250 bps before the ATG of the ORFs to encompass the promoter region.

Three additional pMS82-derived plasmids were constructed containing different parts of the multidomain *SCO4439* gene: one harboring the *SCO4439* N-terminus and the other two harboring two regions of the *SCO4439* C-terminus. The *SCO4439*-N-terminus truncated gene was generated in two steps: first, the whole *SCO4439* gene was amplified with primers B2F/B3R and cloned into pCR™-Blunt II-TOPO^®^, selecting for the plasmid in which the C-terminus of the *SCO4439* gene was orientated to the *Spe*I side of the pCR™-Blunt II-TOPO^®^ (pTOPO4439); second, the DD-CPase domain was deleted by digesting pTOPO4439 with *Nru*I and *Spe*I, the *Spe*I cohesive end was filled with the Klenow DNA polymerase, and the plasmid was religated to generate pTOPO4439-P-N. A stop codon (TAG) from the *Spe*I restriction site was formed in the correct ORF. Two C-terminus constructions were performed, one including the 5′ region of the gene (promoter and RBS) followed by the DD-CPase domain (*SCO4439*-P-C), and the second including the 5′ region followed by both the transmembrane and DD-CPase domains (*SCO4439*-P-T-C). *SCO4439*-P-C was generated by digesting pTOPO4439 with *Afe*I and *Nru*I, and re-ligating the plasmid. pTOPO4439-P-C, lacked the *Afe*I-*Nru*I fragment (the putative transcriptional regulatory and transmembrane domains) but conserved the 5′-region and the open reading frame. *SCO4439*-P-T-C was created in three steps: first the 5′ region was amplified with primers BRB2F/BRB6R and cloned into pCR™-Blunt II-TOPO^®^ (pTOPO4439-P); second, the *SCO4439* C-terminus (including the transmembrane and DD-CPase domains) was amplified with BRB3R/BRB6F and cloned into pCR™-Blunt II-TOPO^®^, and the plasmid in which the *Nde*I (introduced in primer BRB6F) orientated to the *EcoR*V side of the pCR™-Blunt II-TOPO^®^ was selected, to generate pTOPO4439-T-C; and third, the promoter region was released from pTOPO4439-P with *EcoR*V-*Nde*I and cloned into pTOPO-T-C digested with the same enzymes to generate pTOPO4439-P-T-C. pTOPO4439-P-T-C conserved the open reading frame of the transmembrane and DD-CPase domains. The three truncated genes were subcloned into pMS82, generating plasmids pBRB4, pBRB5 and pBRB6 (Table 1).

The seven pMS82-derived plasmids (pBRB1-pBRB6 and pBRB3*) were independently conjugated into the *SCO4439::Tn5062* strain as indicated above. The integration of these plasmids into the pMS82 integration site (gene *SCO4848*)^41^ was verified by PCR using primers SCO4848F (hybridizing with the *SCO4848* gene) and pMS82R (hybridizing with pMS82). Plasmid integration was confirmed via the generation of a 617-bp amplicon.

### Viability staining

Culture samples were obtained and processed for microscopy at various incubation durations, as previously described^42^. The cells were stained with propidium iodide and SYTO 9 (LIVE/DEAD Bac- Light Bacterial Viability Kit, Invitrogen, L-13152). The samples were observed under a Leica TCS-SP8 confocal laser-scanning microscope at wavelengths of 488 nm and 568 nm excitation and 530 nm (green) or 640 nm (red) emissions^42^. More than 30 images were analyzed for each developmental time point in a minimum of three independent cultures. For spore diameter quantification, the images were calibrated with Image J, and the diameter of at least 100 spores was quantified for each strain and developmental time point (Supplementary Figs S1 and S2). These images included pictures from at least three biological replicates.

### Time-lapsed (live) imaging

Initially spores were incubated on GYM medium; after 6 hours of incubation, the samples were cut out and inverted into uncoated m-dishes (Ibidi GmbH). The lid was turned so it was supported on the vents, allowing gas exchange, and sealed off by two layers of Parafilm to prevent medium drying. The samples were incubated at 30 °C and imaged with a Leica TCS-SP8 confocal laser-scanning microscope. Images were taken using the interference contrast mode (unstained samples) every 10 minutes for 12 hours. Time-lapse images were processed with Image J. Time lapse experiments were limited to 12 hours because prolonged incubations dry the culture medium and interfere with hyphal growth.

### RNA extraction and Real-Time Quantitative Reverse Transcription PCR (qRT-PCR)

Total RNA samples from three biological replicates of each developmental time point were obtained. Approximately 100 mg of mycelia (fresh weight) were scraped from the GYM-cellophane medium using a plain spatula. Five hundred microliters of RNA Protect Bacteria reagent (Qiagen) were added to the mycelia to provide immediate RNA stabilization. The extraction was carried out using the RNeasy Mini Kit (Qiagen). The lysis step was made using Fast-Prep (MP™ Biomedicals) with two 30-s force 6.5 cycles, with 1 minute on ice between each run. A phenol acid extraction was performed immediately prior to applying the samples to the column. Treatments with DNase I (Qiagen) and TURBO DNA-freeTM kit (Ambion) were performed to eliminate possible chromosomal DNA contamination. RNA integrity was verified using a 2100 Bioanalyzer (Agilent).

Quantitative RT-PCR (qRT-PCR) was performed as previously described by Yagüe *et al*.^15^. Relative quantification of gene expression was calculated using the (REST) software tool^43^. Primer efficiencies were measured using different dilutions of genomic DNA as templates.

### Resistance of spores to sonication, lysozyme, mild acid, heating and freezing

Freshly prepared suspensions of spores were prepared at a concentration of 10^8^ spores/ml in sterile distilled water, and subjected to different treatments as detailed below. Germination of the spores prior to and after treatment were analyzed by plating and quantifying the number of colony-forming units. All quantifications were performed in triplicate, and the data correspond to the average ± SD of the replicates.

For sonication, 2 ml of spores were treated in an MSE Soniprep (six cycles of 15 seconds of sonication, 1 minute on ice). For lysozyme incubation, 1 ml of spores was treated with a concentration of 50 μg/ml freshly prepared lysozyme (Sigma-Aldrich, L6876) and incubated at 37 °C for 30 minutes. For mild acid treatment, 0.2 ml of spores were incubated with 0.1 N of HCl for 5 minutes at 25 °C; acid was neutralized via 20-fold dilution in 50 mM potassium phosphate buffer (pH 7.1). For heating, the spores were heated at 55 °C for 90 minutes. For freezing, the spores were stored at −20 °C for 24 hours.

### Determination of the minimum inhibitory concentration (MIC)

Minimum inhibitory concentrations (MICs) of teicoplanin and vancomycin (Sigma-Aldrich V1130 and T0578) were determined in GYM by adding increasing concentrations of glycopeptides. The inoculum was 10^5^ spores/plate, and the plates were incubated at 30 °C until colonies appeared. The MIC was the lowest concentration of the antibiotic that inhibited the visible growth of *S. coelicolor*^44^,^45^. The experiments were performed in triplicate, and were highly reproducible with a variation of zero.

### *SCO4439* and SCO4439* gene overexpression and protein purification

The *SCO4439* and SCO4439* (SCO4439 mutated in Leu_684_) genes were amplified with primers BRB7F/BRB7R from pBRB3/pBRB3* and cloned into the expression vector pET11a (Novagen) to generate pBRB7/pBRB7*. A His_6_ tag was included at the 5′-terminus of the BRB7F primer. The SCO4439/SCO4439* genes were overexpressed in *E. coli* JM109 (DE3) using the MagicMedia *E. coli* Expression Medium (Invitrogen K6803). The expression was performed at 18 °C for 36 hours following the manufacturer’s protocol. The cells were harvested via centrifugation, resuspended in buffer A (20 mM sodium phosphate, 0.5 M NaCl, 40 mM imidazole, complete EDTA-free Protease Inhibitor Cocktail Tablets from Roche, pH 7.4) and ruptured using Fast-Prep (MP™ Biomedicals) with ≤106 μm beads (Sigma-Aldrich, G8893) and three 20-s force 6.5 cycles, with 1 minute on ice between each run. Finally, the samples were centrifuged at 7,740 × *g* for 15 minute at 4 °C. The resulting supernatant fraction was centrifuged at 100,000 × g for 1 hour at 4 °C, and the supernatant was used for protein purification.

Recombinant His_6_-SCO4439/ His_6_-SCO4439* were purified using 1 ml HisTrap HP affinity columns from GE Healthcare (reference 17-5247-01). Buffer A, described above, was used as a binding buffer; the elution buffer was similar but contained 500 mM imidazole. The protein was purified using an Amersham Pharmacia FPLC (LCC 500 plus controller and two P500 pumps). Purification was performed at 4 °C using a flow of 1 ml per minute, a 20 ml linear elution gradient, and collecting fractions of 500 μl. Fractions were analyzed via SDS-PAGE Coomassie gels, and those containing the overproduced protein were combined, quantified by Bradford^46^, and used for further experiments.

### Mass spectrometry analysis

The identity of the overproduced protein was confirmed via peptide mass fingerprinting. The overproduced purified His_6_-SCO4439 protein was manually excised from a 1D Coomassie gel, and the proteins were digested following the method of Havlis *et al*.^47^, and analyzed using a 4800 Proteomics Analyzer matrix-assisted laser desorption ionization time-of-flight (MALDI-TOF/TOF) mass spectrometer (AB Sciex). Protein identification, was performed using Mascot v. 2.2.04.

### Assay of DD-dipeptidase and DD-carboxypeptidase activities

Enzymatic activities were assayed as reported previously^35^,^45^ by measuring the release of D-Ala from commercially available dipeptide (D-Ala-D-Ala, 10 mM; Sigma-Aldrich, A0912) and tripeptide (N_α_,N_ε_-diacetyl-l-Lys-D-Ala-D-Ala, 10 mM; Sigma-Aldrich, D9904) in the reaction buffer (100 mM Tris-HCl, pH 7.2), together with different amounts of the purified recombinant His_6_-SCO4439 or His_6_-SCO4439*. The release of D-Ala was followed spectrophotometrically with a D-amino acid oxidase (Sigma-Aldrich, A5222)-peroxidase (Sigma-Aldrich 77332) coupled reaction that oxidized the colorimetric substrate 4-aminoantipyrine (Sigma-Aldrich 06800) to chinonemine in the presence of phenol solution (Sigma-Aldrich P4557)^35^,^48^. One unit of enzyme activity is defined as the amount of enzyme that produced 1 μmol D-Ala per minute from the tripeptide as substrate; this value must be halved when the dipeptide is used as substrate. To measure the inhibition of DD-CPase/DD-dipeptidase activity, the protein was incubated with increasing concentrations (from 0 to 5 mM) of penicillin G (Sigma-Aldrich, P3032) for 15 minutes at 37 °C and then added to the assay mixtures. All measurements were performed in triplicate, and the data correspond to the means ± SD.

### Cellular fractioning

*S. coelicolor* and the *SCO4439::Tn5062* mutant were grown in solid GYM medium as described above for 16, 30 and 72 hours at 30 °C. The mycelium collected from cellophane discs were suspended in 2 ml of 0.9% NaCl per gram of cells (wet weight). All of the following manipulations were carried out at 0 to 4 °C, and all solutions contained proteinase inhibitors (0.19 mg/ml phenylmethanesulfonyl fluoride and 0.7 g/ml pepstatin, both purchased from Sigma-Aldrich P7626 and P5318), unless otherwise stated. Mycelia were fragmented by sonication with a Sonics Vibra-Cell VCX 130. Sonication was carried out for 5 minutes on ice with cycles of 30 seconds with an amplitude of 90% (90% of 60 Hz), and breaks of 10 seconds. The samples were then centrifuged at 39,000 × *g* for 15 minutes, and the supernatants (cytoplasmic fractions) were collected. Alkaline extraction of the insoluble fraction (membranes and cell wall debris) was carried out by adapting a previously developed protocol for extracting membrane-bound proteins in enterococci by Kariyama *et al*.^49^ and recently adapted to *Streptomyces* by Binda *et al*.^43^. The sedimented pellets were resuspended in ice-cold distilled water; immediately prior to centrifugation (28,000 × *g* for 15 minutes at 4 °C), the pH was adjusted to 12 by adding an appropriate volume of 2.5 N NaOH. Immediately after centrifugation, the supernatants (resuspended insoluble fractions) were neutralized to pH 7 by adding 0.5 M sodium acetate (pH 5.4)^35^,^36^,^45^. Enzymatic activities in the cytosolic fractions and the re-suspended insoluble fractions were assayed as previously reported^36^,^45^.

### FDNB determination of spore PG crosslinking

The protocol described by Atrih *et al*.^50^ to analyze the PG crosslinking in the spores of *Bacillus* was adapted to *S. coelicolor*. The protocol for PG extraction was modified as follows: spores were collected from solid SFM media^38^; the concentration of spores used for extraction was 3 mg (dry weight) per ml of extraction buffer; FDNB treatment was performed using 200 μl of the extracted spore cell walls. This protocol works for the analysis of the PG from *Streptomyces* spores, but not for mycelium, perhaps due to the difficulty to homogenize the dense pellets of the mycelium, making PG poorly accessible to the extraction and FDNB treatment.

Glycine and diaminopimelic acid (Dpm) were measured via high-performance liquid chromatography using pre-column derivatization with *o*-phthaldehyde (OPA) and UV detection (338 nm). The chromatographic equipment used included the Agilent 1100 HPLC System: a G1312A binary pump, G1329A autosampler, and G1315B-Diode Array Detector. Data collection and integration were performed using Software Chem Station LC 3D. The column used was a 250 × 3.9 mm, 100 Å, Symmetry C18 (5 μm) (WAT046980) from Waters. The binding buffer (10 mM Na_2_HPO_4_, 40 mM boric acid, pH 8.15) and the elution buffer (MeOH:ACN:H_2_O; 45:45:10, v/v/v) were filtered (0.45 μm) prior to use. Samples were eluted in an increasing gradient of elution buffer (20% for 1.9 minutes, 70% for 13 minutes; 100% for 2.7 minutes) with a flow of 1ml /minute. The column temperature was 40 °C, the injection volume 20 μl, and the detection of the amino acids was at 338 nm. Pure glycine and Dpm (both from Sigma-Aldrich) were used as standards.

The crosslinking index defined by Atrih *et al*.^50^ is based on the difference between the Dpm measured in the FDNB-treated and untreated samples. FDNB treatment is performed prior to PG hydrolysis and blocks NH groups of the Dpm residues that have not formed crosslinks. NH groups blocked with FDNB cannot react with the derivative reagent used for HPLC UV detection. *S. coelicolor* differs from *Bacillus*, and PG crosslinking is formed by Gly instead of Dpm^51^. Consequently, in this work, the crosslinking index was calculated as the ratio between the cross-linked Gly (Gly detected in the FDNB-treated samples) and total Gly (Gly detected in the non-treated samples). Dpm was used to normalize the glycine measurements (expressed as a ratio to Dpm).

### Bioinformatic analyses

Transmembrane topology of the SCO4439 gene was analyzed using Phobius software (http://phobius.sbc.su.se/).

Orthologous sequences to SCO4439 from other streptomycetes were obtained from the databases at the National Center for Biological Information (http://www.ncbi.nlm.nih.gov). The accession numbers of the selected sequences were: WP_016326920 (*S. lividans*), NP_824958 (*S. avermitilis*), YP_006878621 (*S. venezuelae*), WP_013002845 (*S. scabies*), YP_001825771 (*S. griseus*), and WP_003961441 (*S. clavuligerus*). The DD-CPase domains of the proteins were aligned using MUSCLE software, and amino acid similarities were estimated by using Lalign software (http://www.ch.embnet.org/software/LALIGN_form.html).

### Fluorescent vancomycin staining

Nascent PG synthesis was stained in *Streptomyces* liquid cultures growing in GYM medium^37^, inoculated with spores at a concentration of 1 × 10^7^ viable spores/ml, and incubated at 30 °C and 200 rpm for 5 and 8 hours. The samples were stained as previously described^20^. BODIPY-vancomycin (Invitrogen V34850) was mixed with an equal amount of unlabeled vancomycin (Sigma SBR00001). The vancomycin and BODIPY-vancomycin mixtures were added to the cultures at final concentrations of 1 μg/ml and incubated for 20 minutes. Cells were fixed for 15 minutes at room temperature using PBS (0.14 M NaCl, 2.6 mM KCl, 1.8 mM KH_2_PO_4_ and 10 mM Na_2_HPO_4_) containing 2.8% paraformaldehyde and 0.0045% glutaraldehyde, and observed under a Leica TCS-SP8 confocal laser scanning microscope at 505 nm excitation and 513 nm emission wavelengths.

## Additional Information

**How to cite this article**: Rioseras, B. *et al*. Characterization of SCO4439, a D-alanyl-D-alanine carboxypeptidase involved in spore cell wall maturation, resistance, and germination in *Streptomyces coelicolor*. *Sci. Rep.* **6**, 21659; doi: 10.1038/srep21659 (2016).

## Supplementary Material

Supplementary Information

Supplementary Movie 1

Supplementary Movie 2

Supplementary Movie 3

Supplementary Movie 4

## Figures and Tables

**Table 1:** Bacterial strains, plasmids and primers used in this study.

Strain, plasmid, cosmid	Description	Reference
Bacterial strains		
*S.coelicolor* M145	SCP1^−^ SCP2^−^	38
S.*coelicolor SCO4439::Tn5062*	*SCO4439*-40::Tn5062, Am^R^	This study
*E. coli* TOP10	F- mcrA Δ(mrr-hsdRMS-mcrBC) φ80lacZΔM15 ΔlacX74 nupG recA1 araD139 Δ(ara-leu)7697 galE15 galK16 rpsL(Str^R^) endA1 λ^−^	Invitrogen
*E. coli* JM109 (DE3)	*E. coli* JM109 containing a chromosomal copy of the gene for T7 RNA polymerase	Promega
*E. coli* ET12567		
*E. coli* ET12567/pUZ8002	*E. coli* ET12567 containing plasmid pUZ8002, a not self-transmissible plasmid which can mobilize other plasmids	53
Plasmids & cosmids		
pQM5062	Plasmid containing *eGFP* Tn5062	40
pMS82	Cloning vector, Hyg^R^	41
pBRB1	SCO4440-4442 harbouring its own promoter cloned into pMS82/*SpeI*/*EcoRV*, Hyg^R^	This study
pBRB2	SCO4437-4439 harbouring its own promoter cloned into pMS82/*SpeI*/*EcoRV*, Hyg^R^	This study
pBRB3	*SCO4439* harbouring its own promoter cloned into pMS82/*Spe*I/*EcoR*V, Hyg^R^	This study
pBRB3*	*SCO4439* harbouring its own promoter containing a mutation (Leu_684_ was changed by Pro) and cloned into pMS82/*Spe*I/*EcoR*V, Hyg^R^	This study
pCR™-Blunt II-TOPO^®^	Zero Blunt^®^TOPO^®^PCR Cloning Kit, Km^R^	Invitrogen
pTOPO4439	*SCO4439* harbouring its own promoter cloned into pCR™-Blunt II-TOPO^®^, Km^R^	This study
pTOPO4439-P-N	pTOPO4439 digested with *Nru*I and *Spe*I (filled blunt) and religated, Km^R^	This study
pTOPO4439-P-C	pTOPO4439 digested with *Afe*I and *Nru*I and religated, Km^R^	This study
pTOPO4439-P	*SCO4439* promoter cloned into Blunt TOPO, Km^R^	This study
pTOPO4439-T-C	Transmembrane region and carboxyl end of *SCO4439* cloned into pCR™-Blunt II-TOPO^®^, Km^R^	This study
pTOPO4439-P-T-C	pTOPO4439-P digested with EcoRV/NdeI and cloned in pTOPO-4439-T-C digested with *EcoR*V/*Nde*I, Km^R^	This study
pBRB4	Fragment *EcoR*V/*Hind*III from pTOPO4439-P-N cloned in *Hind*III and *Kpn*I (filled blunt) of vector pMS82, Hyg^R^	This study
pBRB5	Fragment *EcoRV*/*SpeI* from pTOPO4439-P-C cloned in *EcoR*V/*Spe*I of pMS82/*Spe*I/*EcoR*V, Hyg^R^	This study
pBRB6	Fragment *EcoR*V/*Spe*I from TOPO4439-P-T-C cloned in *EcoR*V/*Spe*I of pMS82/*Spe*I/*EcoR*V, Hyg^R^	This study
pET11a	Cloning/expression vector, Ap^R^	Novagen
pBRB7	His-SCO4439 cloned in pET11a/*Nde*I/*BamH*I, Ap^R^	This study
pBRB7*	His-SCO4439 containing a mutation (Leu_684_ was changed by Pro) cloned in pET11a/*Nde*I/*BamH*I, Ap^R^	This study
D6.2.B06	D6 cosmid carrying D6.2.B06 transposant	18
Primers		
BRB1F	5′AAAAAGATATCGTCTCGCGGACCGACAGC 3′	This study
BRB1R	5′CCCACTAGTAACTGGTCGAGAGGGCTCC 3′	This study
BRB2F	5′AAAAAGATATCACCGAGGTCGAGCGACTG 3′	This study
BRB2R	5′CCCACTAGTGCTCACCAGCGACAATGAGG 3′	This study
BRB3R	5′CCCACTAGTGTTTGGCGACGCTAGCAC 3′	This study
BRB6R	5′AACATATGCGCCAGCCGCGGATCAC 3′	This study
BRB6F	5′AACATATGACCACCCAGCAGCCGCTG 3′	This study
BRB7F	5′TTCATATGCATCATCATCATCATCATGTGGCCCGGGAGGACG 3′	This study
BRB7R	5 GGGGATCCGTTTGGCGACGCTAGCAC 3′	This study
SCO4848F	5′ CGTCGTATCCCCTCGGTTG 3′	This study
pMS82R	5′ GAGCCGGGAAAGCTCATTCA 3′	This study
SCO4439-qRTPCR-F	5′ GGCGTTCGTGGAGAAGATG 3′	This study
SCO4439-qRTPCR-R	5′ CTCACCGTCGTGTTGTTCAG 3′	This study

The pairs of primers used to amplify fragments cloned in plasmids pBRB1-7 and pTOPO4439-P-N-T-C are described in the Materials and Methods section.
