# Supplementary material for: Characterization of SCO4439, a D-alanyl-D-alanine carboxypeptidase involved in spore cell wall maturation, resistance, and germination in Streptomyces coelicolor
Source: Sci Rep. 2016 Feb 12;6:21659. doi: 10.1038/srep21659 (PMC4751497; doi:10.1038/srep21659)

Characterization of SCO4439, a D-alanyl-D-alanine carboxypeptidase involved in spore cell wall maturation, resistance, and germination in *Streptomyces coelicolor*

Beatriz Rioseras[1], Paula Yagüe[1], María Teresa López-García[1], Nathaly Gonzalez-Quiñonez[1], Elisa Binda[2,3], Flavia Marinelli[2,3] & Angel Manteca,[1*]

[1] Área de Microbiología, Departamento de Biología Funcional and IUOPA, Facultad de Medicina, Universidad de Oviedo, 33006 Oviedo, Spain.
[2] Department of Biotechnology and Life Sciences, University of Insubria, via J. H. Dunant 3, 21100 Varese, Italy.
[3] "The Protein Factory" Research Center, Politecnico of Milano, ICRM CNR Milano and University of Insubria, 21100Varese, Italy.

Supplementary Fig. S1. Master images used to quantify spore diameters in *S. coelicolor* SCO4439::Tn5062, and SCO4439::Tn5062[pBRB3*].

Supplementary Fig. S2. Master images used to quantify spore diameters in *S. coelicolor* [pMS82] and SCO4439::Tn5062[pBRB3].

Supplementary Movie 1. Time-lapse confocal microscopy of the germination of spores from the *S. coelicolor* wild type strain.

Supplementary Movie 2. Time-lapse confocal microscopy of the germination of spores from the *SCO4439::Tn5062* mutant strain.

Supplementary Movie 3. Time-lapse confocal microscopy of the germination of spores from the *SCO4439::Tn5062* [pBRB3] complemented mutant.

Supplementary Movie 4. Time-lapse confocal microscopy of the germination of spores from the *SCO4439::Tn5062* mutant harbouring the *SCO4439** mutated gene (SCO4439::Tn5062[pBRB3*]).

Fig. S1. MASTER IMAGES USED TO QUANTIFY SPORE DIAMETER

Streptomyces coelicolor 5-hours. Spore diameters measured are labelled by lines.

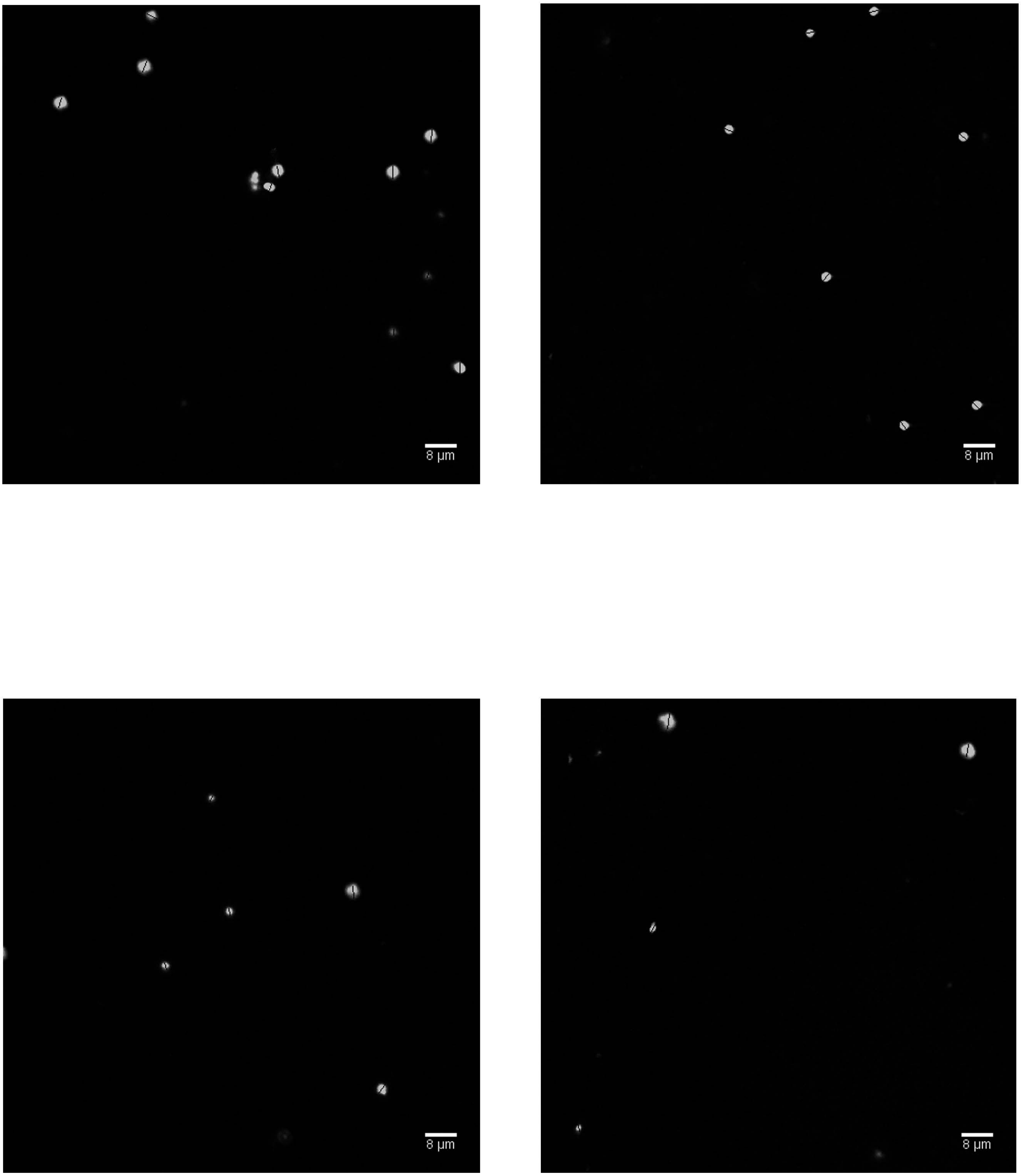

Fig. S1. MASTER IMAGES USED TO QUANTIFY SPORE DIAMETER

Streptomyces coelicolor 5-hours. Spore diameters measured are labelled by lines.

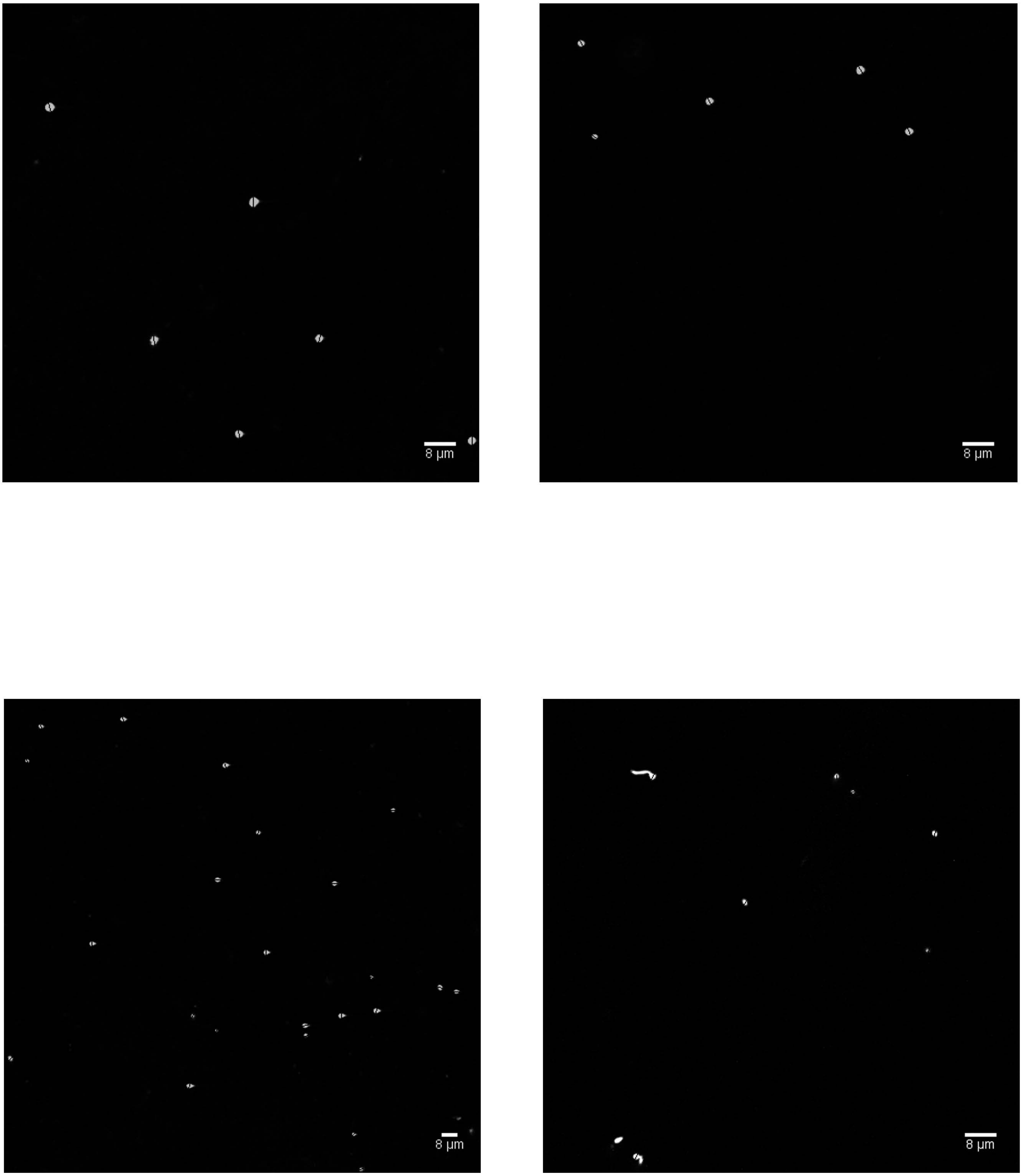

Fig. S1. MASTER IMAGES USED TO QUANTIFY SPORE DIAMETER

Streptomyces coelicolor 5-hours. Spore diameters measured are labelled by lines.

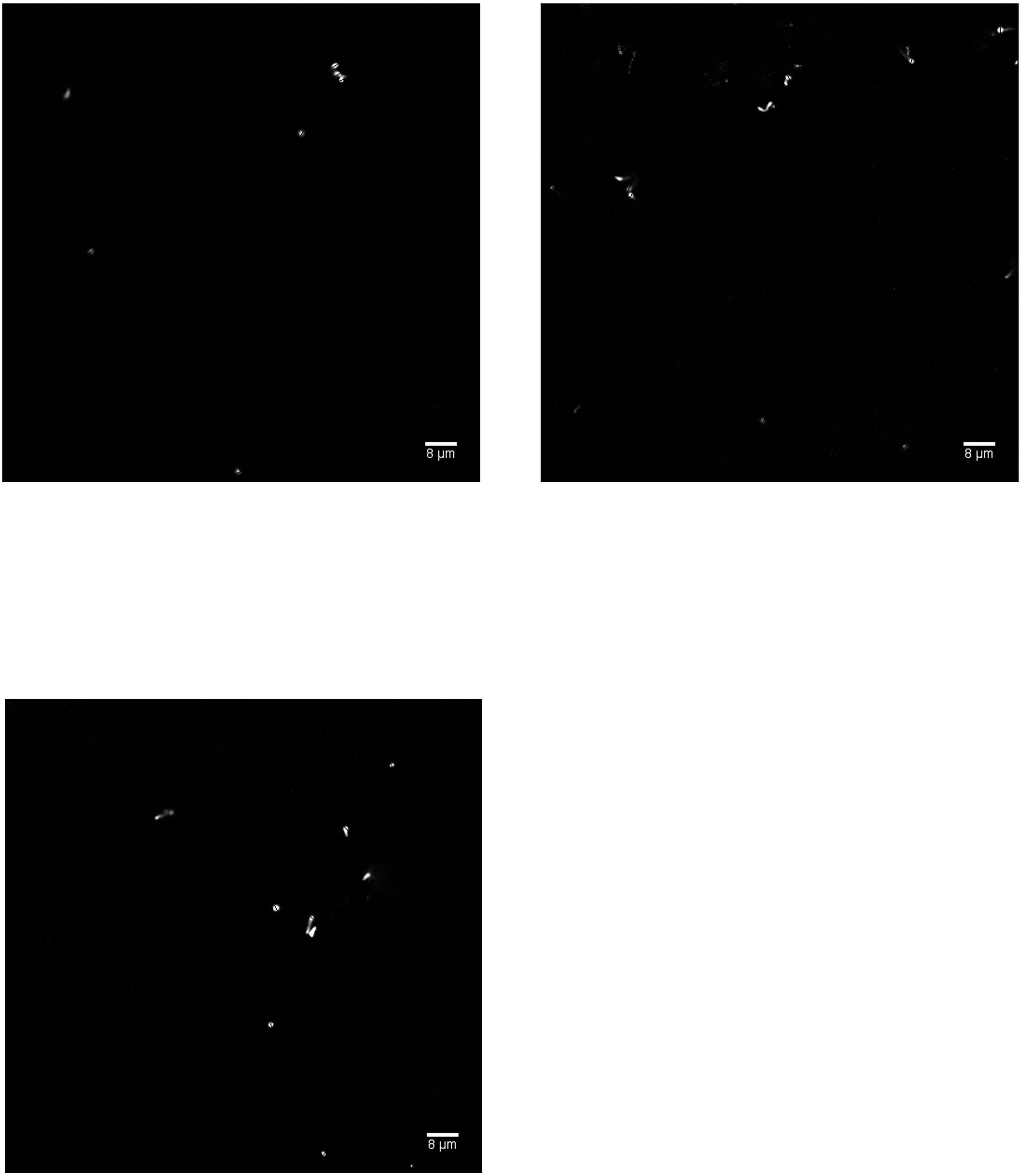

Fig. S1. MASTER IMAGES USED TO QUANTIFY SPORE DIAMETER

Streptomyces coelicolor wild type. 8-hours. Spore diameters measured are labelled by lines.

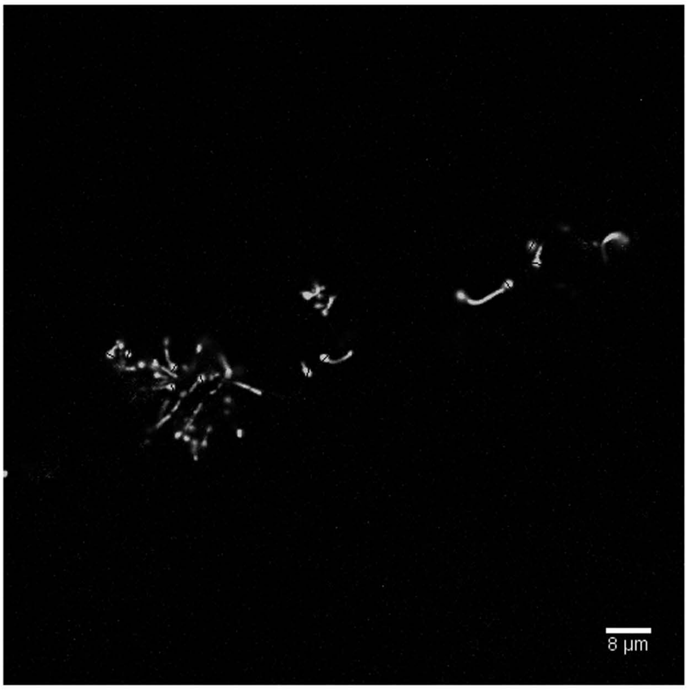

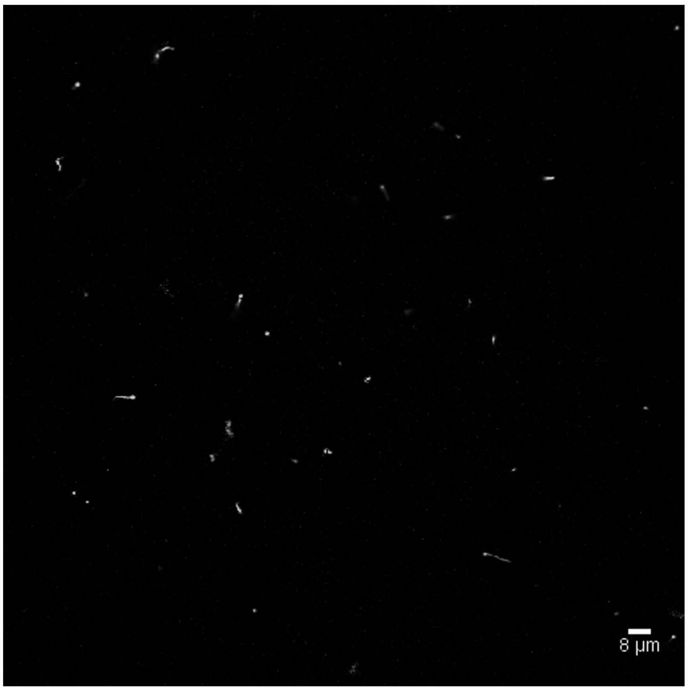

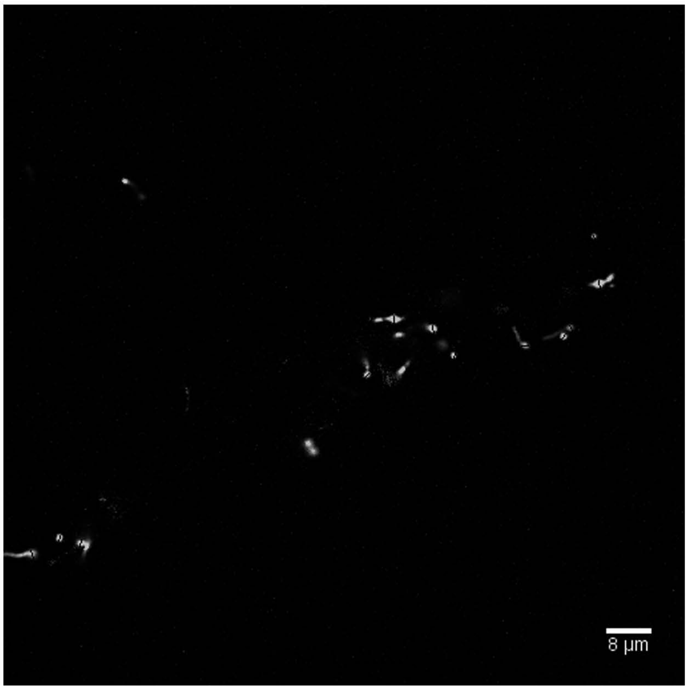

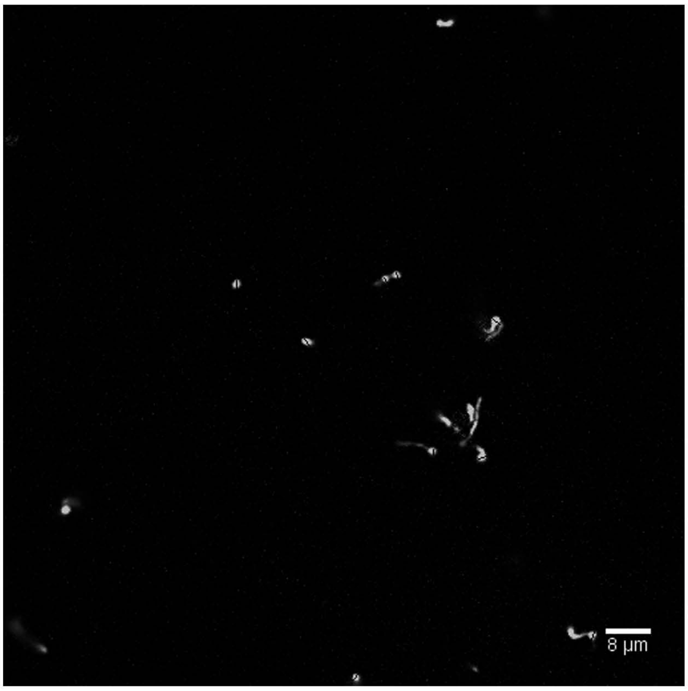

Fig. S1. MASTER IMAGES USED TO QUANTIFY SPORE DIAMETER

Streptomyces coelicolor wild type. 8-hours. Spore diameters measured are labelled by lines.

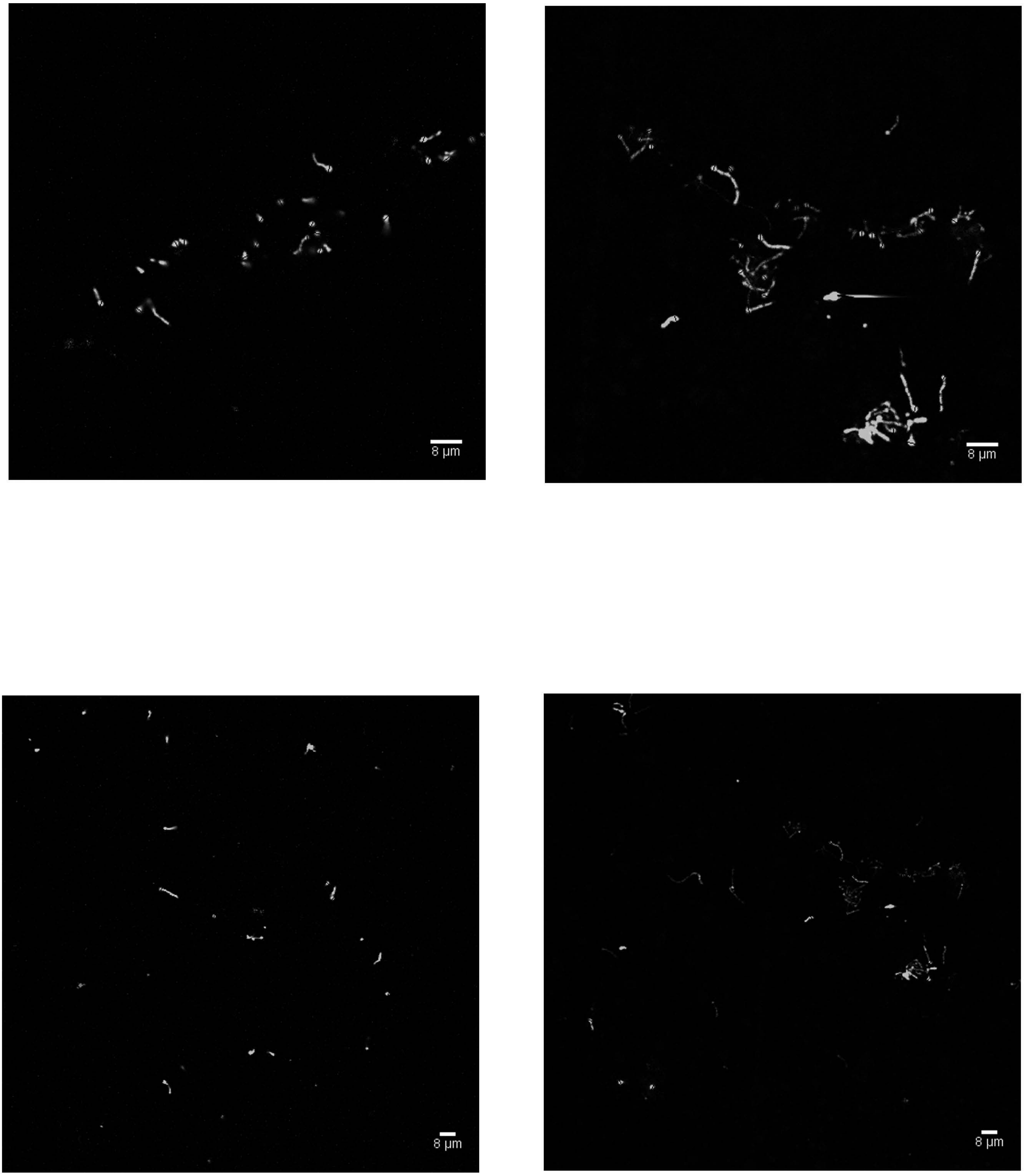

Fig. S1. MASTER IMAGES USED TO QUANTIFY SPORE DIAMETER

Streptomyces coelicolor wild type. 15-hours. Spore diameters measured are labelled by lines.

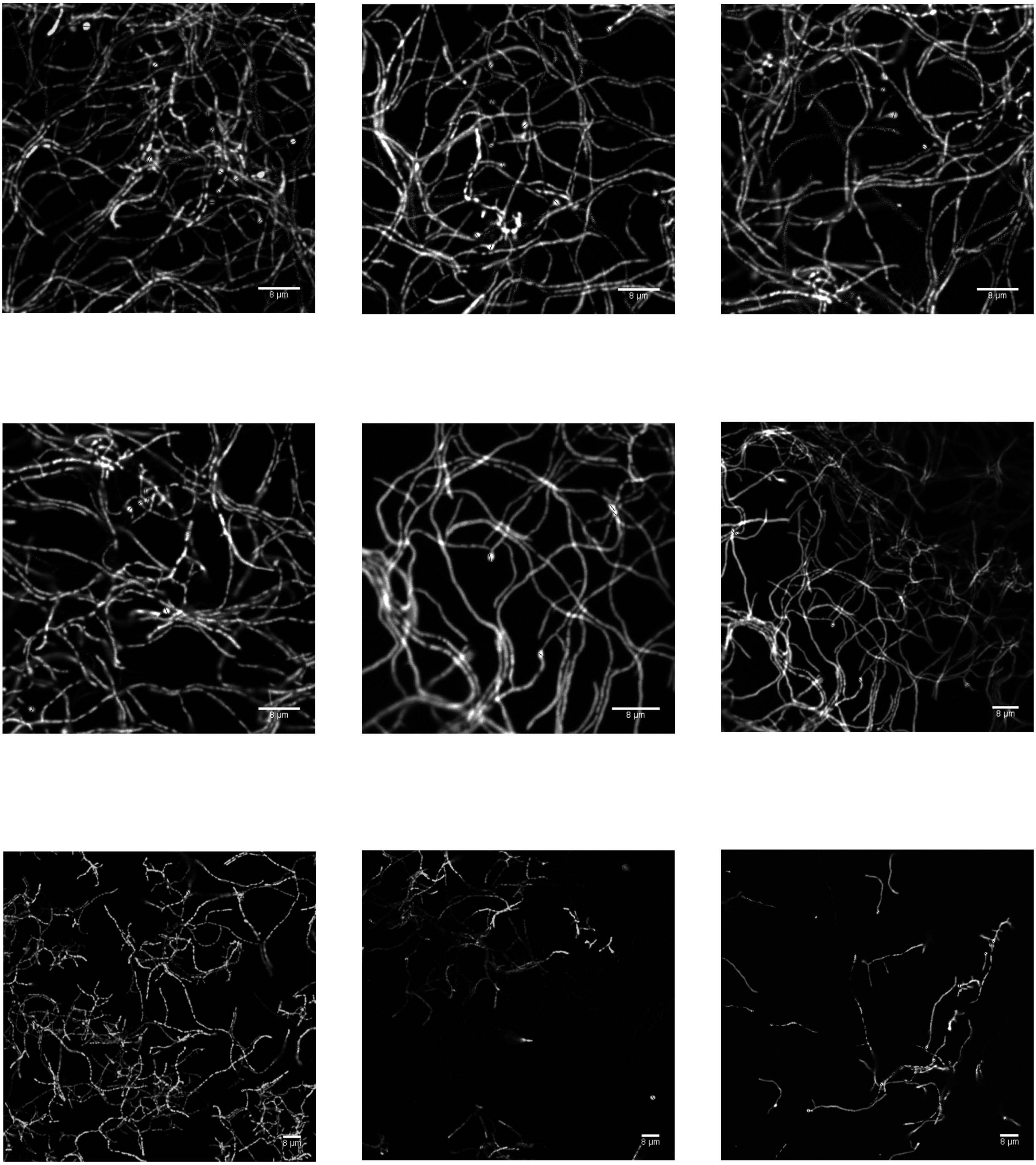

Fig. S1. MASTER IMAGES USED TO QUANTIFY SPORE DIAMETER

Streptomyces coelicolor wild type. 15-hours. Spore diameters measured are labelled by lines.

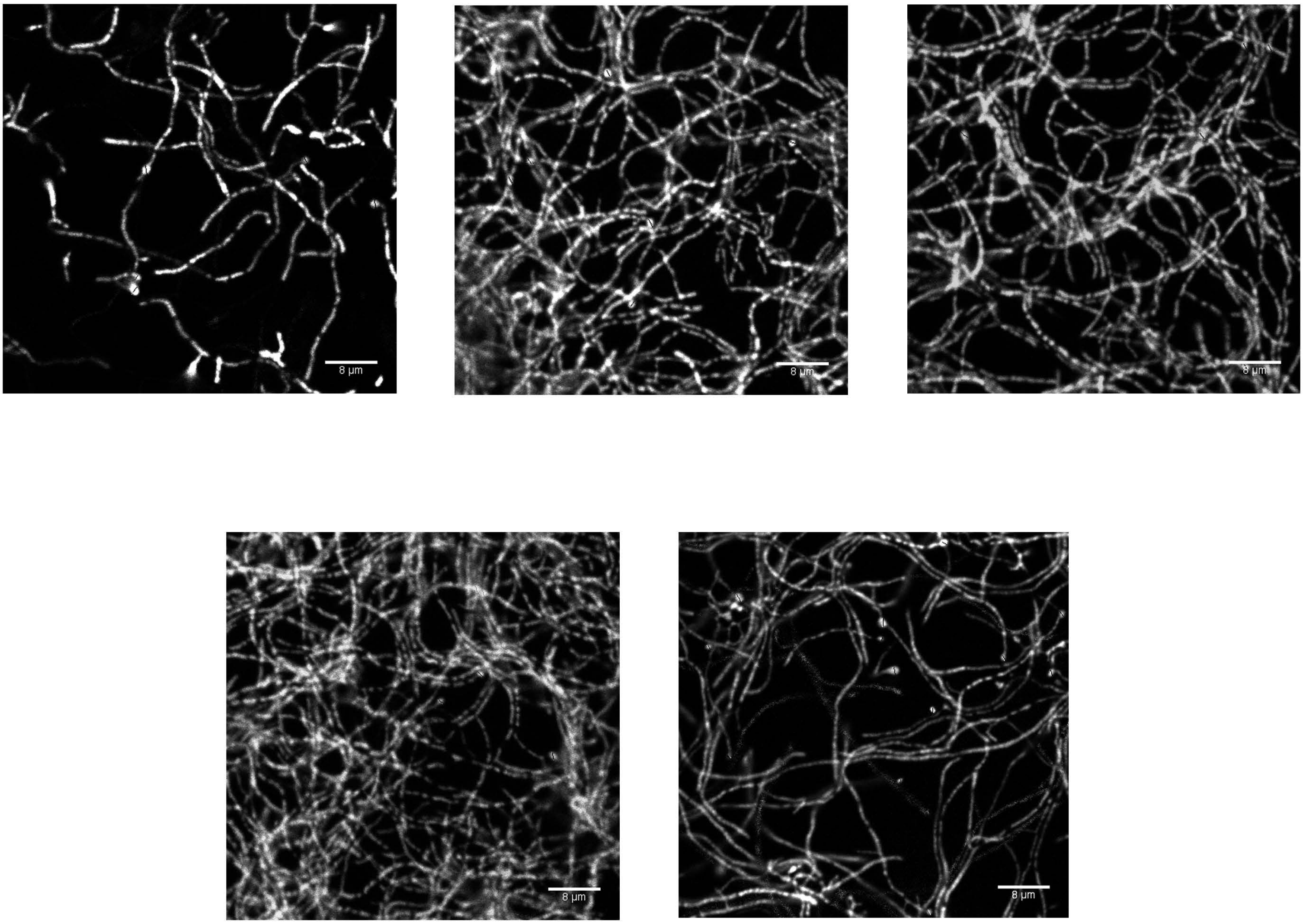

Fig. S1. MASTER IMAGES USED TO QUANTIFY SPORE DIAMETER

Streptomyces coelicolor::Tn5062 5-hours. Spore diameters measured are labelled by lines.

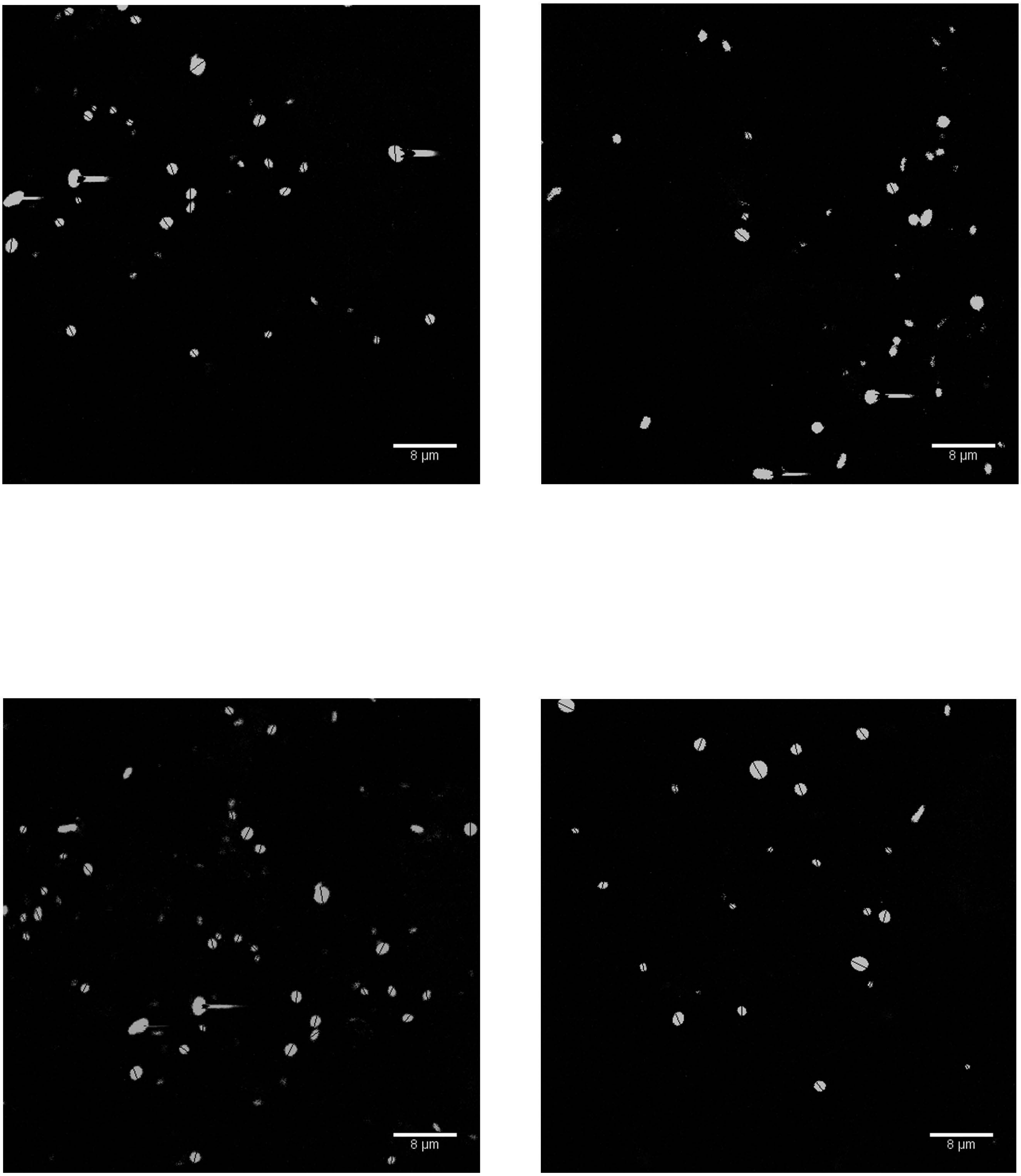

Fig. S1. MASTER IMAGES USED TO QUANTIFY SPORE DIAMETER

Streptomyces coelicolor::Tn5062 5-hours. Spore diameters measured are labelled by lines.

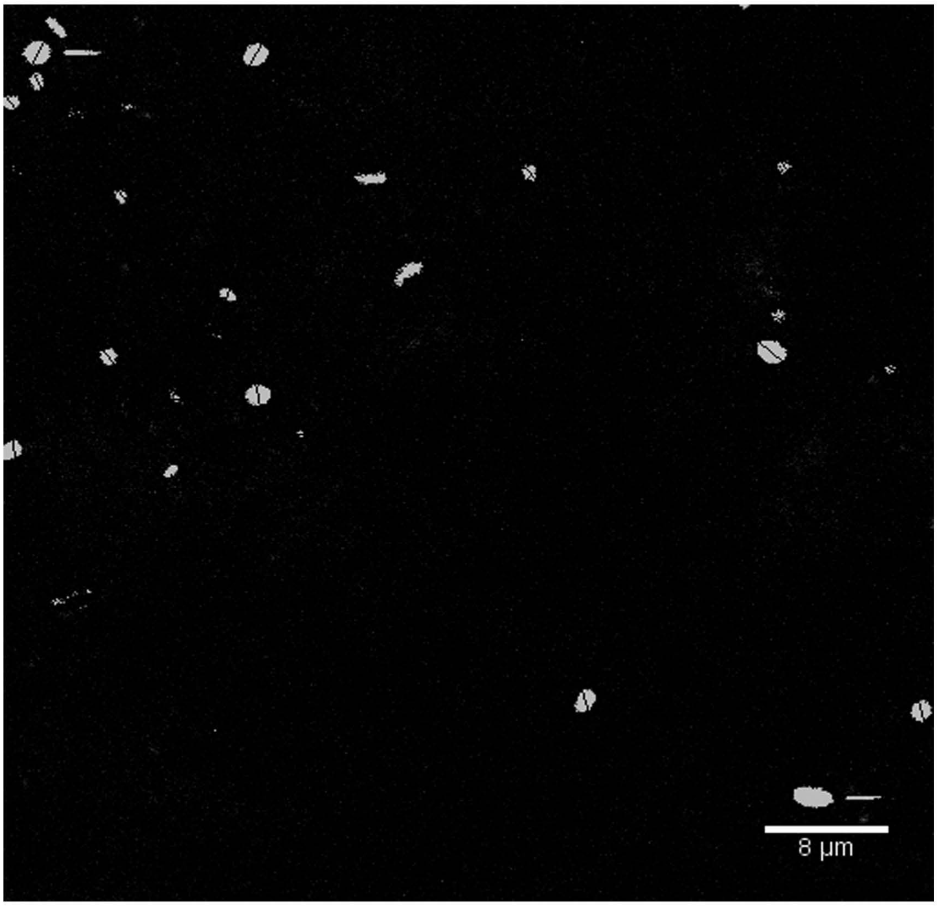

Fig. S1. MASTER IMAGES USED TO QUANTIFY SPORE DIAMETER

Streptomyces coelicolor::Tn5062 8-hours. Spore diameters measured are labelled by lines.

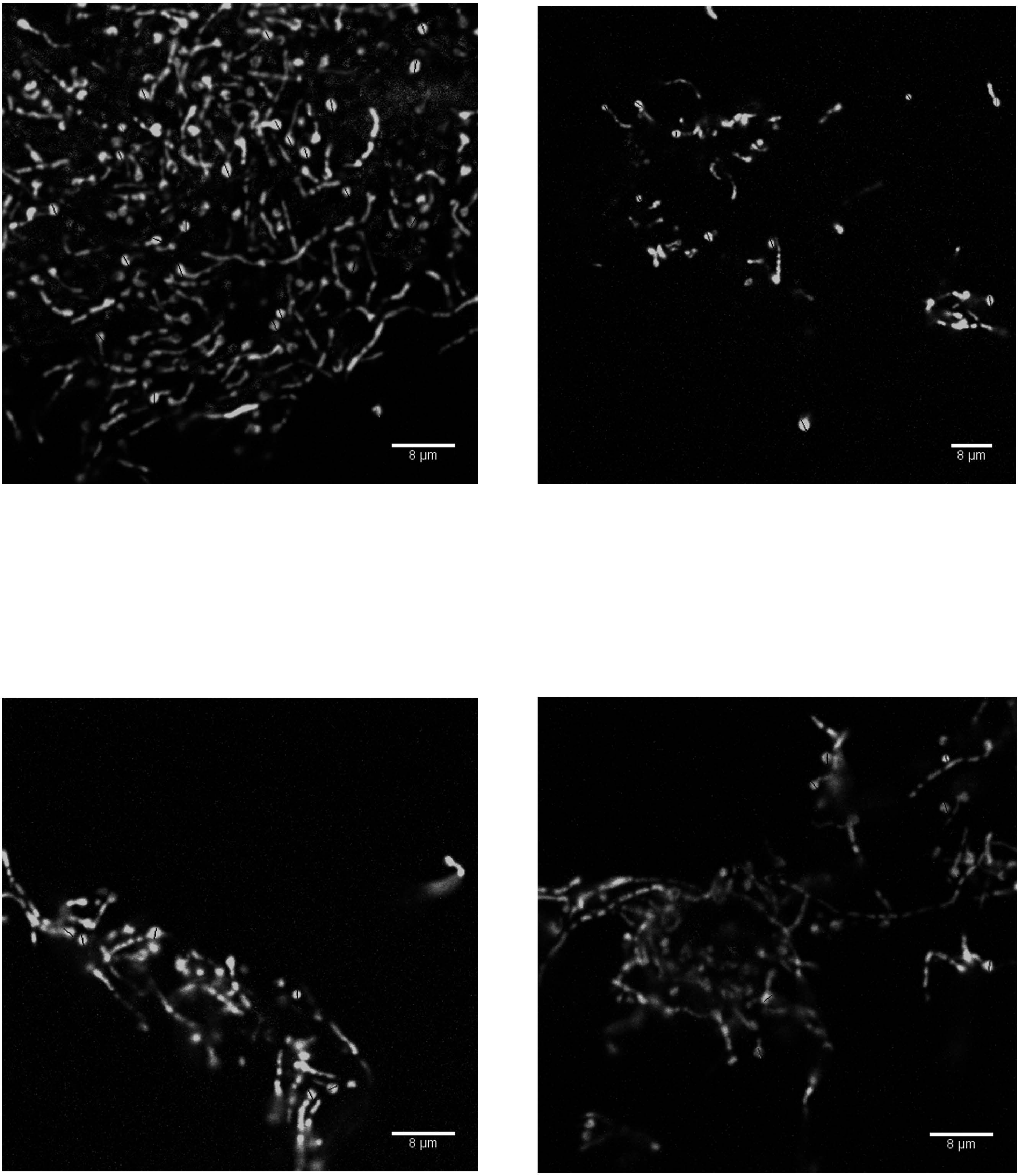

Fig. S1. MASTER IMAGES USED TO QUANTIFY SPORE DIAMETER

Streptomyces coelicolor::Tn5062 8-hours. Spore diameters measured are labelled by lines.

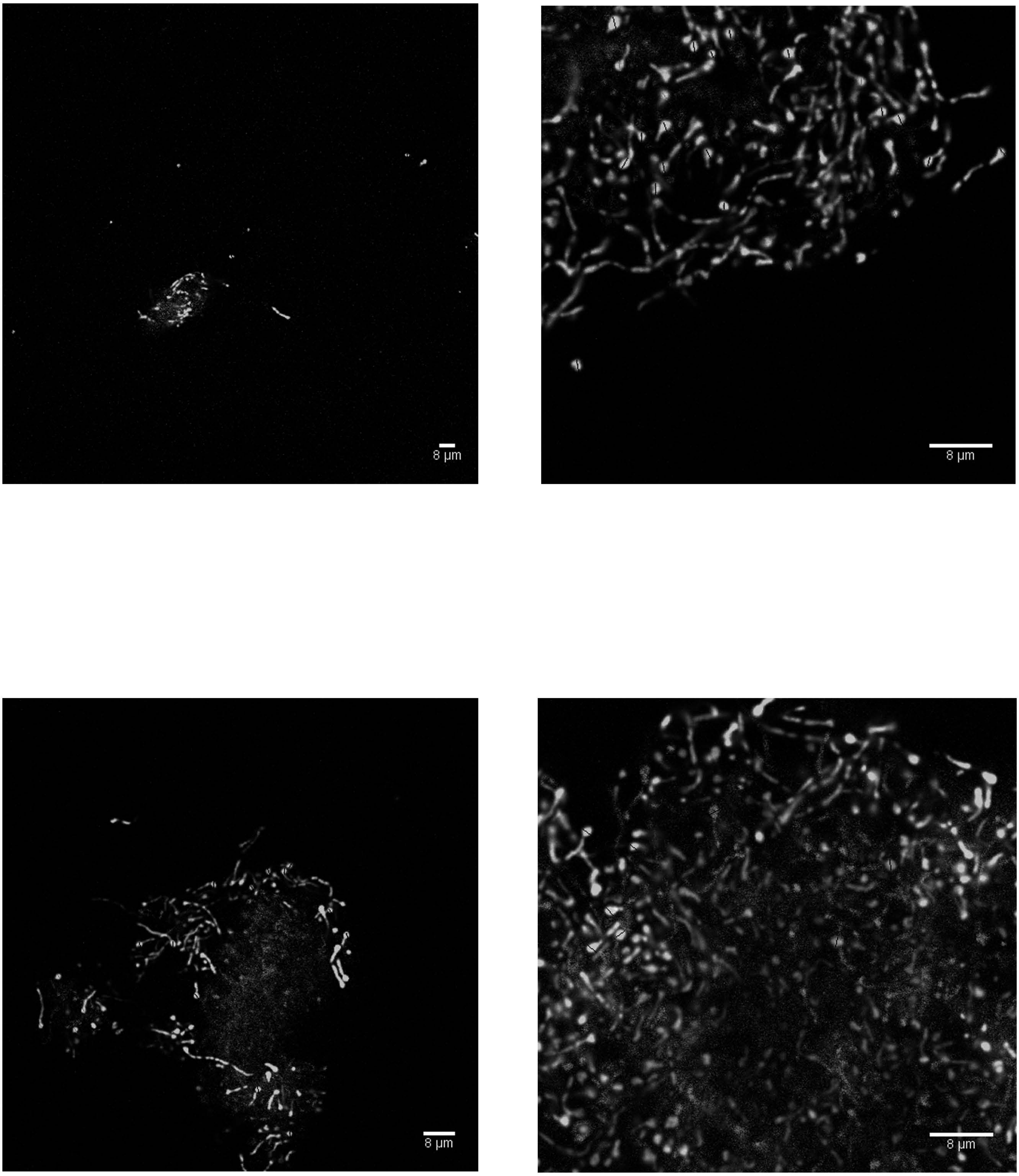

Fig. S1. MASTER IMAGES USED TO QUANTIFY SPORE DIAMETER

Streptomyces coelicolor SCO4439::Tn5062. 15-hours. Spore diameters measured are labelled by lines.

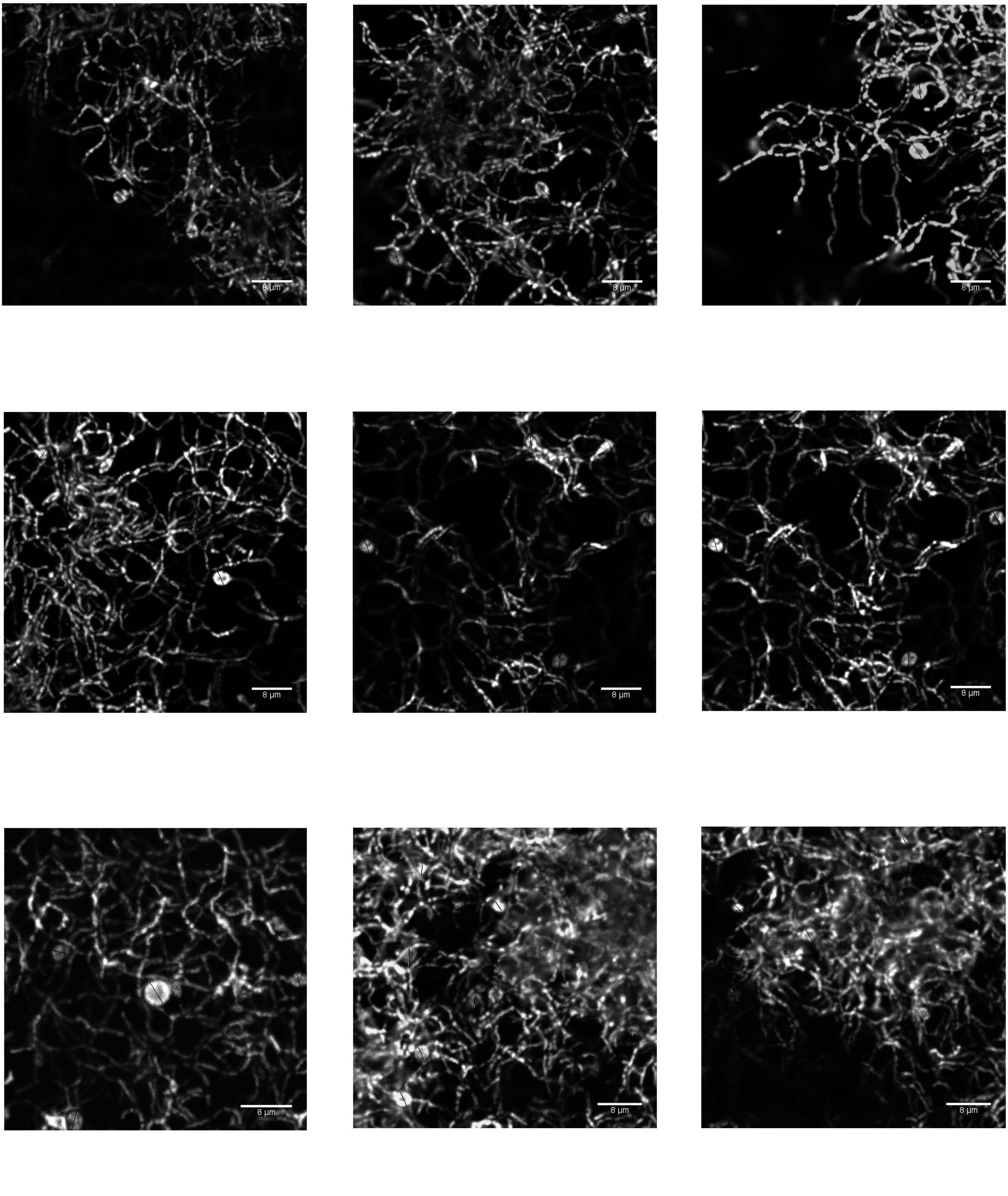

Fig. S1. MASTER IMAGES USED TO QUANTIFY SPORE DIAMETER

Streptomyces coelicolor SCO4439::Tn5062. 15-hours. Spore diameters measured are labelled by lines.

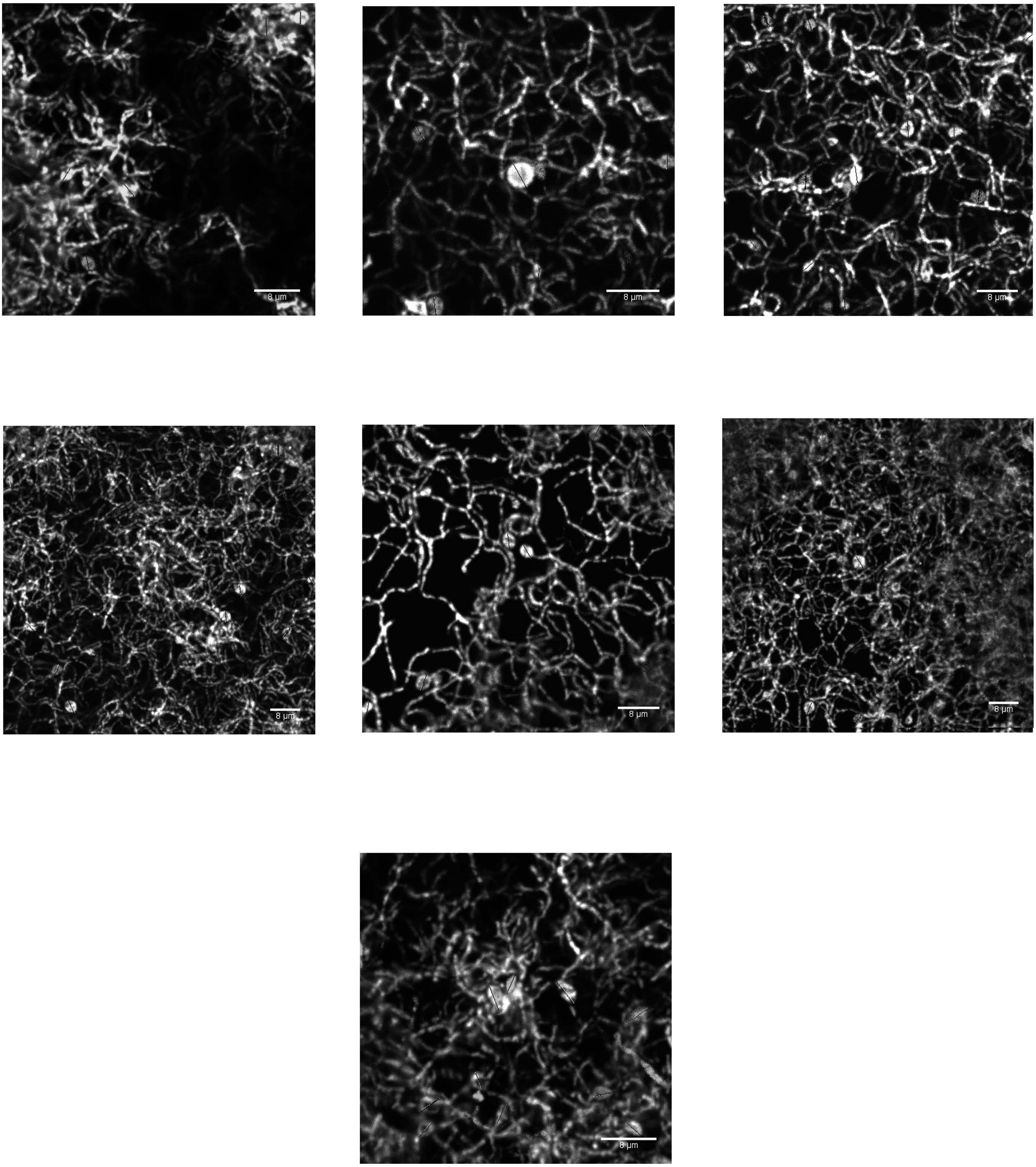

Fig. S1. MASTER IMAGES USED TO QUANTIFY SPORE DIAMETER

Streptomyces coelicolor::Tn5062[pBR3*] 5-hours. Spore diameters measured are labelled by lines.

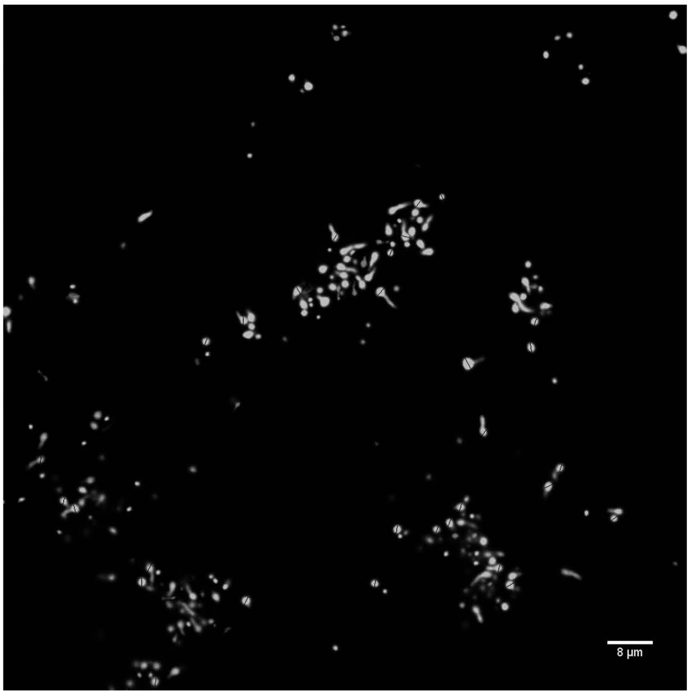

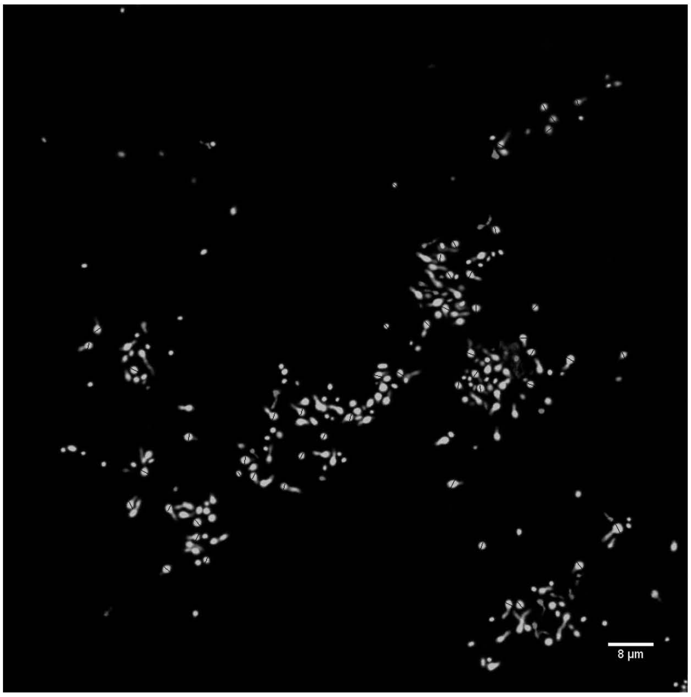

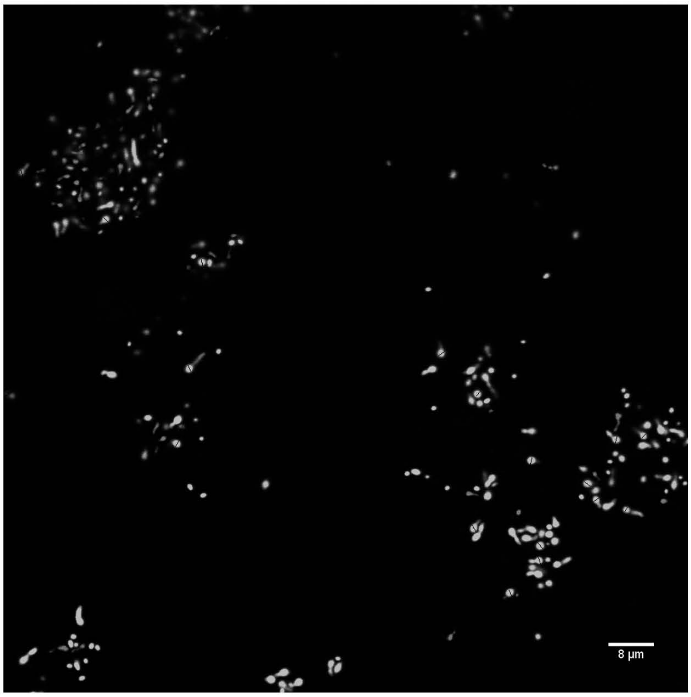

Fig. S1. MASTER IMAGES USED TO QUANTIFY SPORE DIAMETER

Streptomyces coelicolor::Tn5062[pBR3*] 8-hours. Spore diameters measured are labelled by lines.

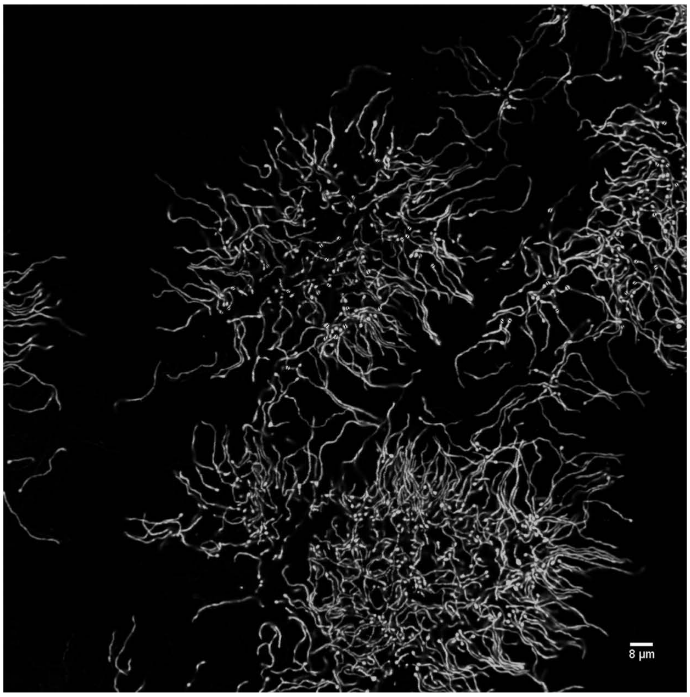

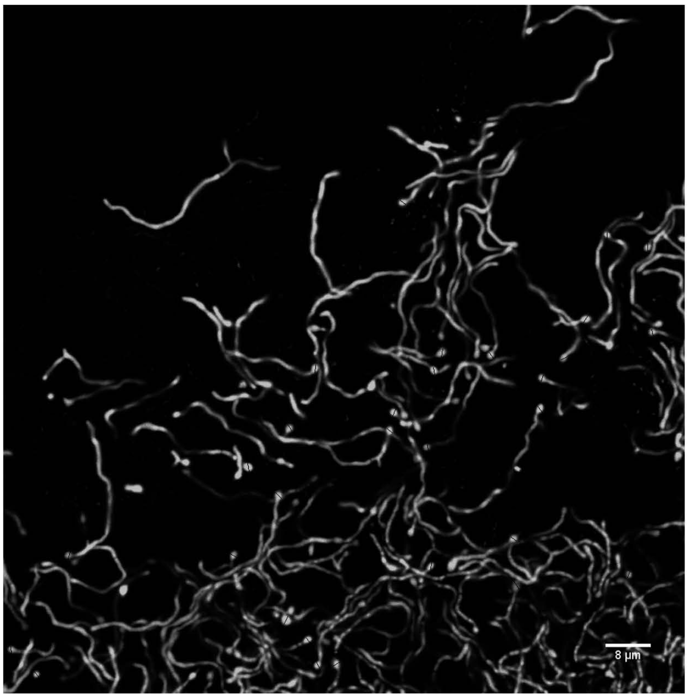

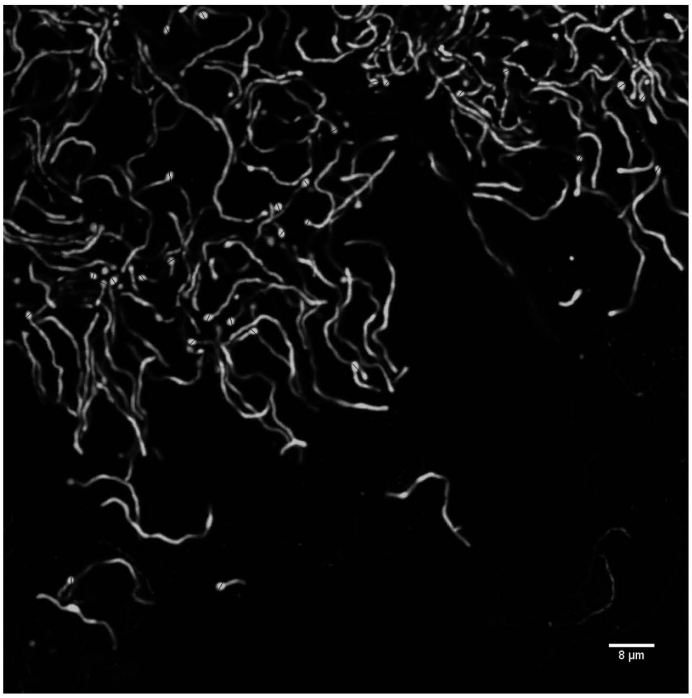

Fig. S1. MASTER IMAGES USED TO QUANTIFY SPORE DIAMETER

Streptomyces coelicolor::Tn5062[pBR3*] 15-hours. Spore diameters measured are labelled by lines.

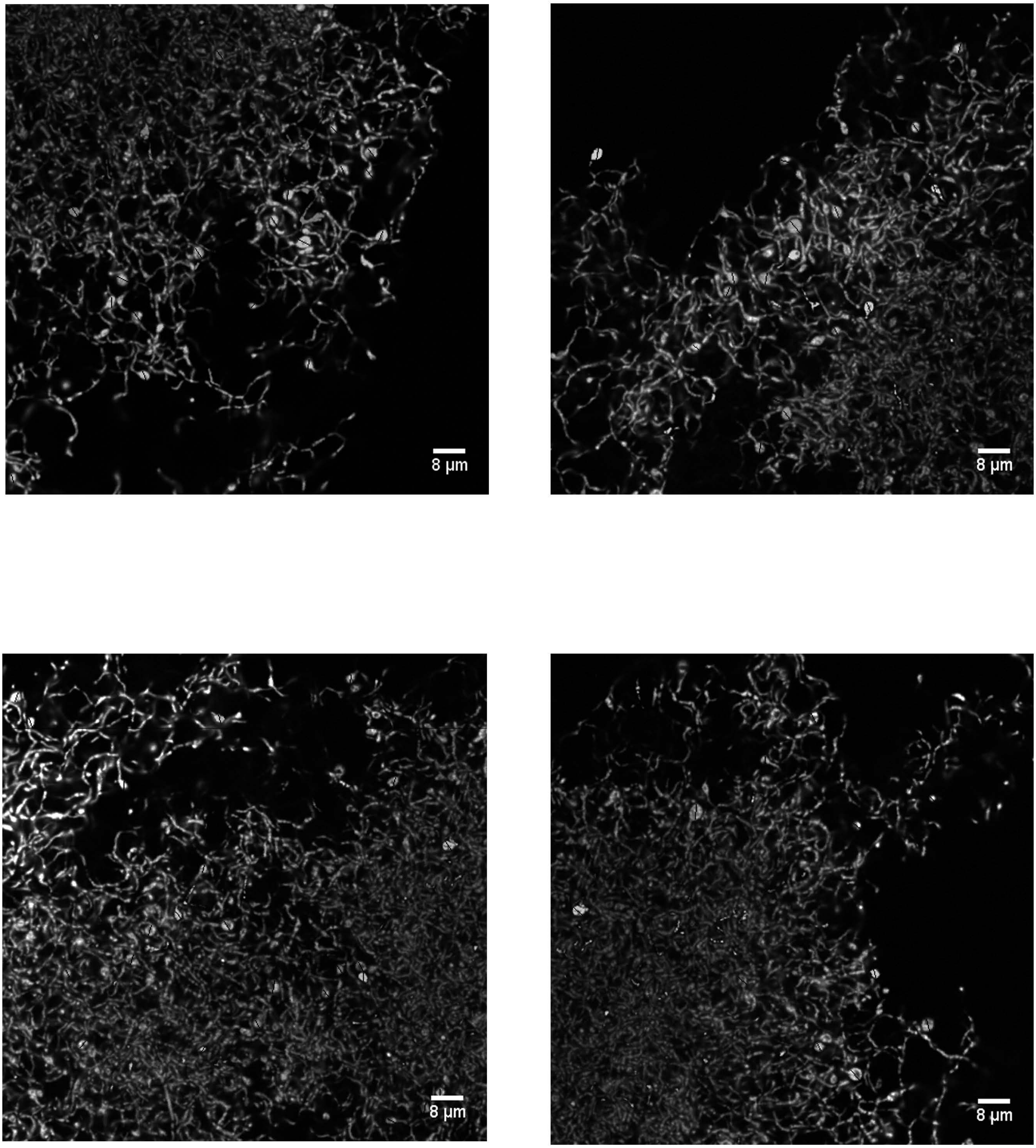

Fig. S1. MASTER IMAGES USED TO QUANTIFY SPORE DIAMETER

Streptomyces coelicolor::Tn5062[pBR3*] 15-hours. Spore diameters measured are labelled by lines.

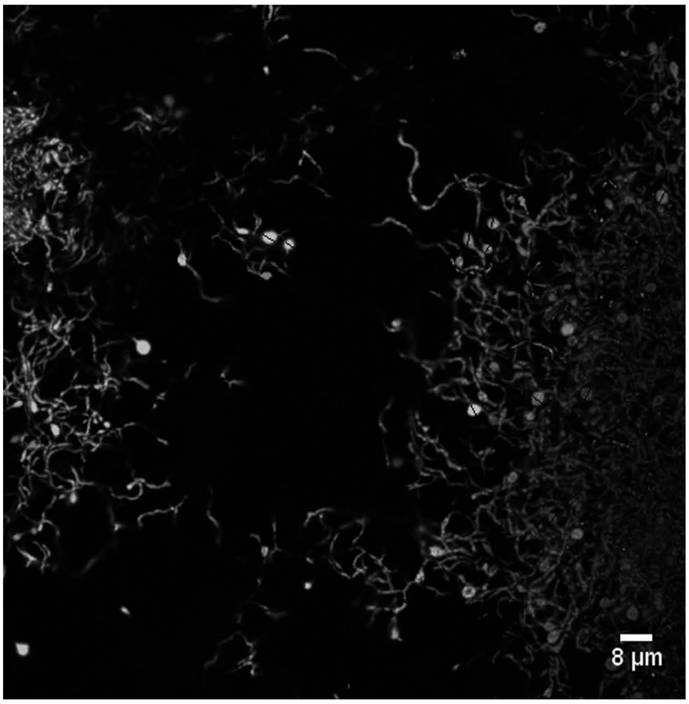

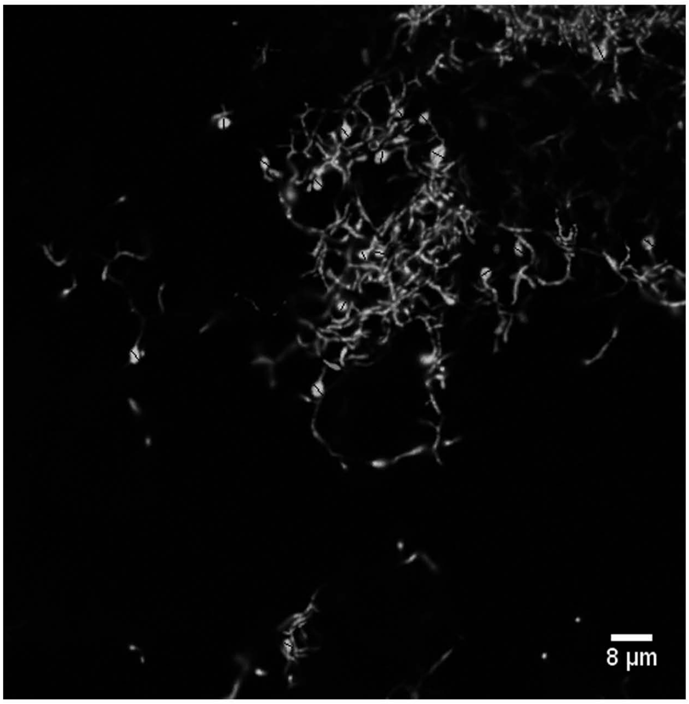

Fig. S2. MASTER IMAGES USED TO QUANTIFY SPORE DIAMETER

Streptomyces coelicolor [pMS82]. 15-hours. Spore diameters measured are labelled by lines.

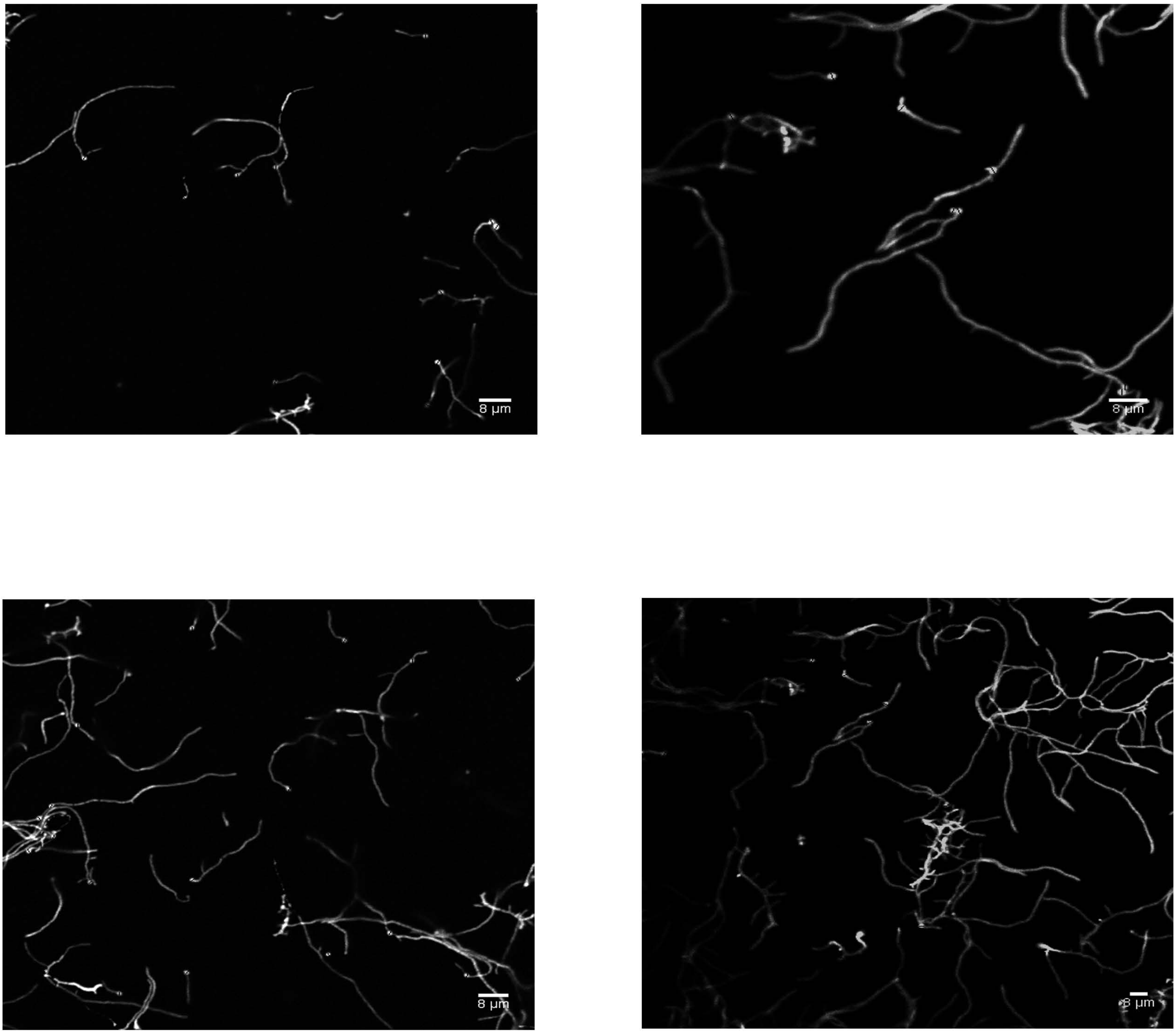

Fig. S2. MASTER IMAGES USED TO QUANTIFY SPORE DIAMETER

Streptomyces coelicolor [pMS82]. 15-hours. Spore diameters measured are labelled by lines.

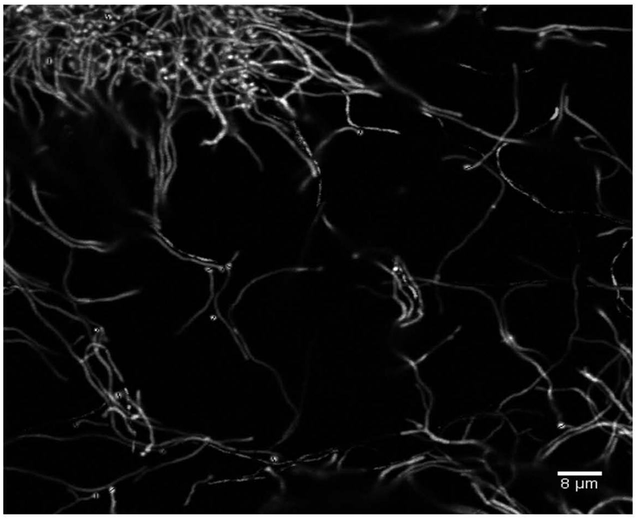

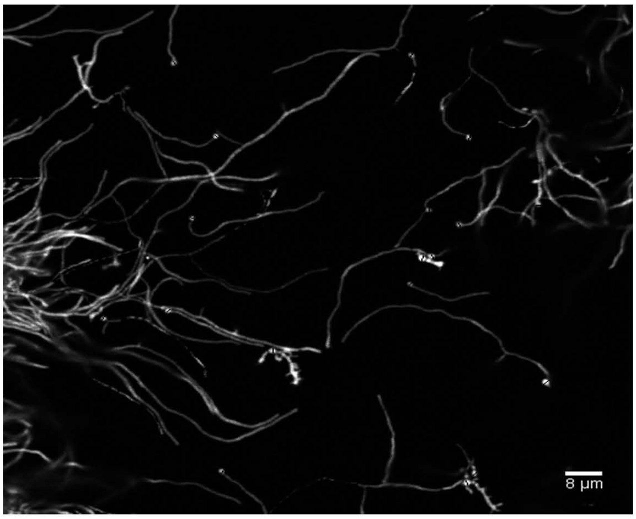

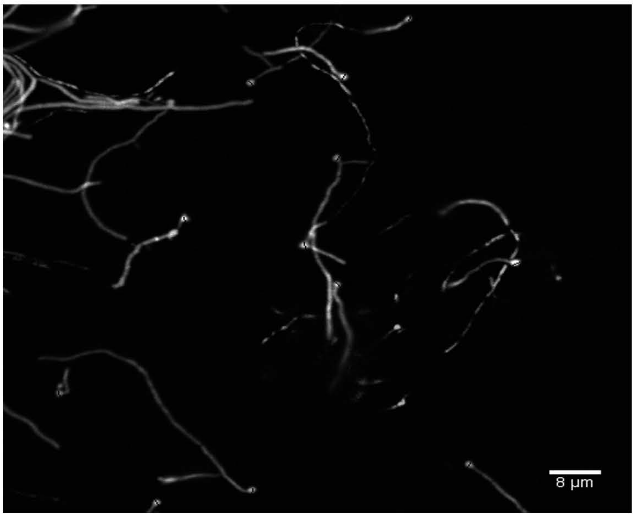

Fig. S2. MASTER IMAGES USED TO QUANTIFY SPORE DIAMETER

SCO4439::Tn5062 [pBRB3]. 15-hours. Spore diameters measured are labelled by lines.

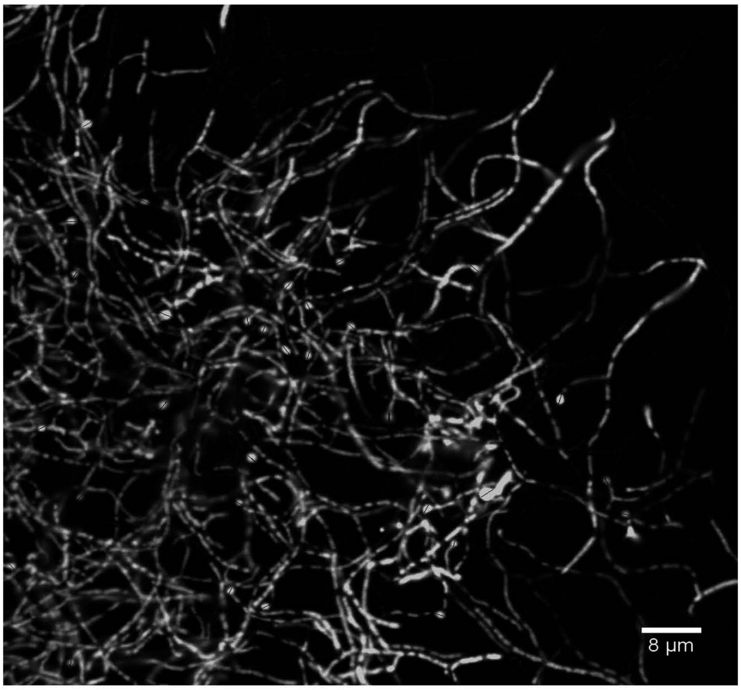

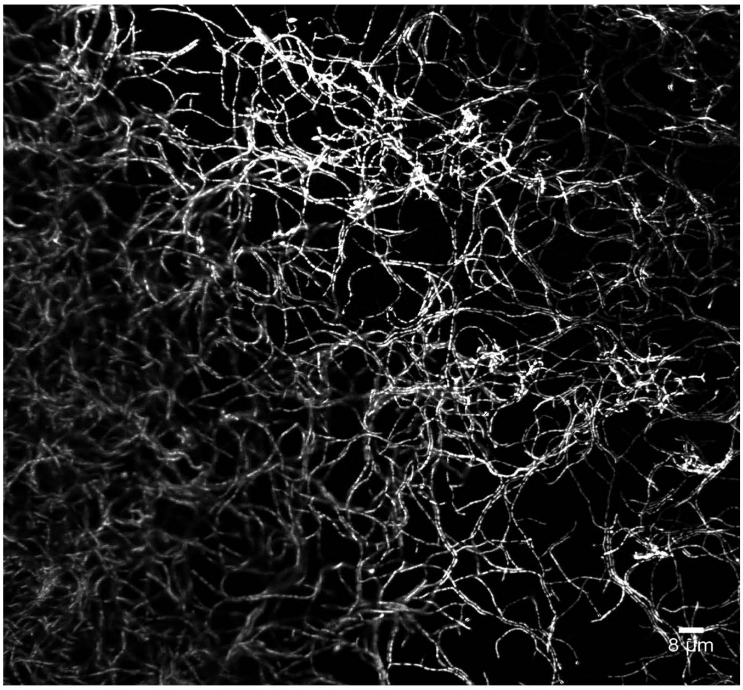

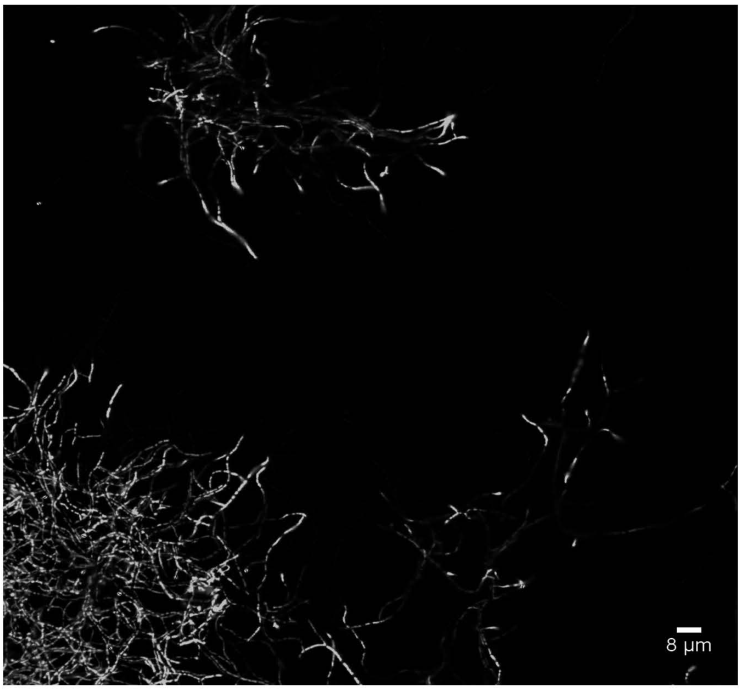

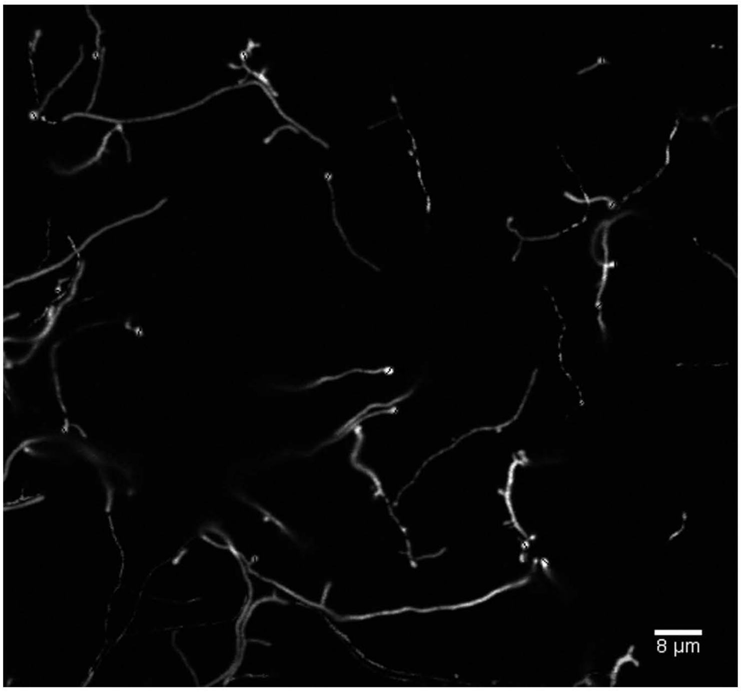

Fig. S2. MASTER IMAGES USED TO QUANTIFY SPORE DIAMETER

SCO4439::Tn5062 [pBRB3]. 15-hours. Spore diameters measured are labelled by lines.

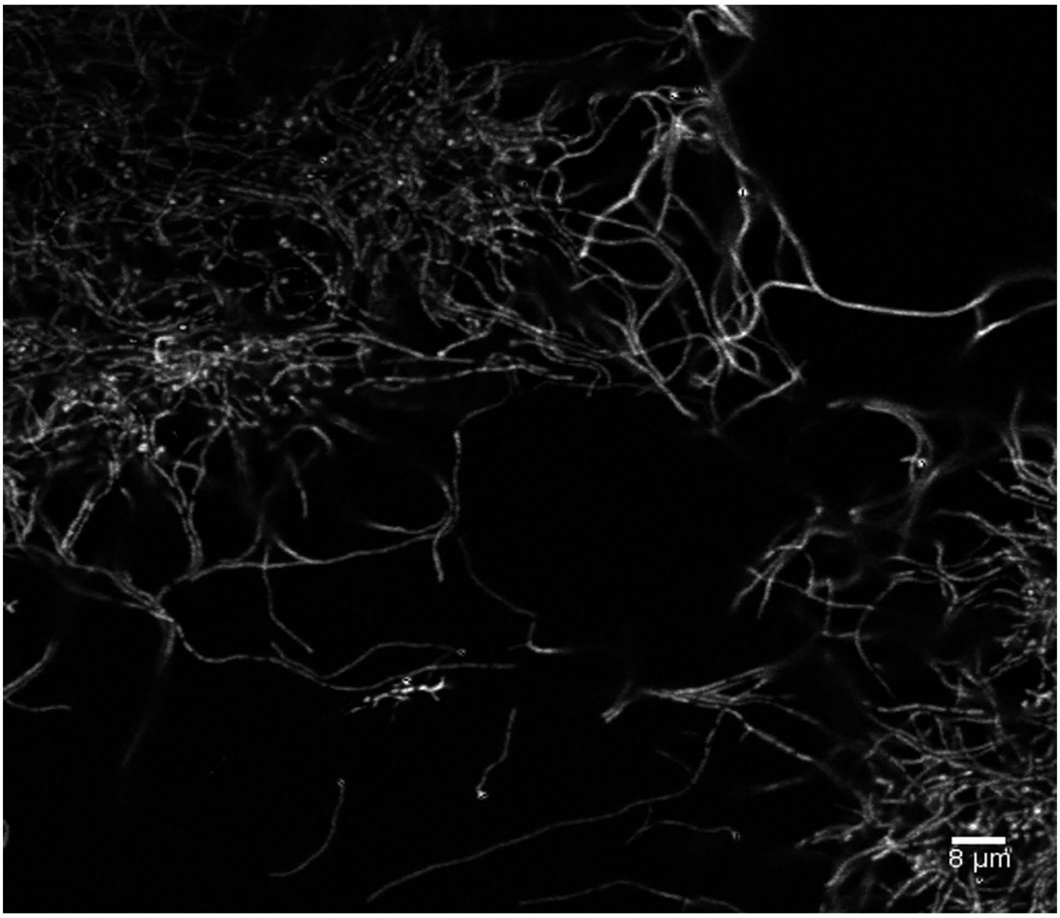

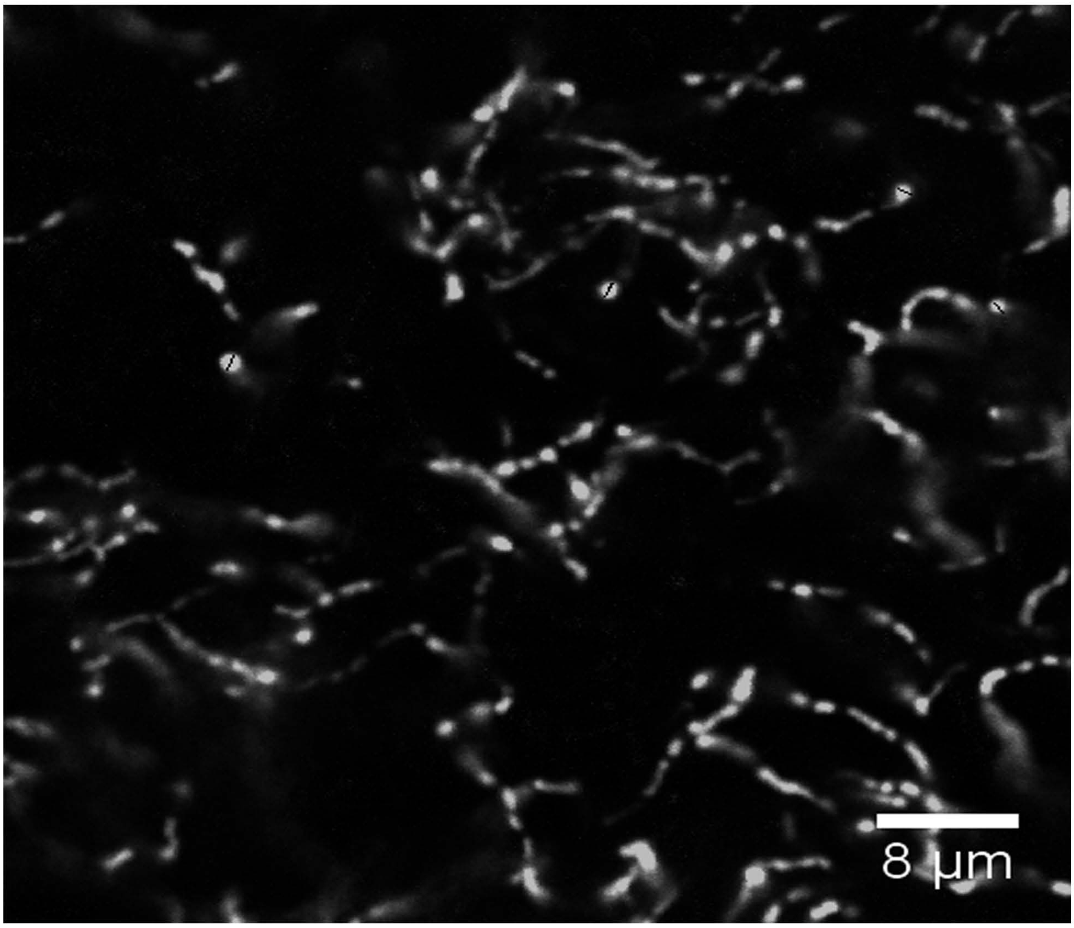

Supplement: Supplementary Information [file srep21659-s1.pdf]
